# High HBV antigen levels drive loss of vaccine-induced CD8^+^ T-cell function in carrier mice over time

**DOI:** 10.1016/j.jhepr.2026.102033

**Published:** 2026-09-10

**Authors:** Edanur Ates Öz, Jinpeng Su, Merve Gültan, Margaret Tulessin, Nikolas Müller, Hélène Anne Kerth, Swati Singh, Carolin Mogler, Katja Steiger, Jochen Wettengel, Dirk Busch, Percy Knolle, Ulrike Protzer, Anna Kosinska

**Affiliations:** 1 Institute of Virology, School of Medicine and Health, Technical University of Munich/Helmholtz Munich, Munich, Germany; 2 German Center for Infection Research (DZIF), Partner Site Munich, Munich, Germany; 3 Institute of Pathology, School of Medicine and Health, Technical University of Munich, Munich, Germany; 4 Institute for Medical Microbiology, Immunology and Hygiene, Technical University of Munich, Munich, Germany; 5 Institute of Molecular Immunology and Experimental Oncology, School of Medicine and Health, Technical University of Munich, TUM University Hospital Rechts der Isar, Munich, Germany

**Keywords:** HBV-specific CD8^+^ T cells, Chronic hepatitis B, CD8 T-cell dysfunction, Therapeutic vaccination, *TherVacB*, Antigen-induced T-cell dysfunction

## Abstract

**Background & Aims:**

Chronic HBV infection persists because of the dysfunction of virus-specific CD8^+^ T cells. High HBV antigen levels impair the success of therapeutic vaccination. The influence of viral titers on vaccine-induced immunity remains to be fully elucidated. We compared the kinetics of vaccine responses in low- and high-titer HBV carrier mice to identify tipping points and dissect the decisive steps determining the outcome of therapeutic vaccination.

**Methods:**

We established low- and high-titer persistent HBV replication in the livers of immunocompetent C57BL/6J mice using adeno-associated-virus (AAV)-HBV and administered *TherVacB*, a heterologous protein prime/modified vaccinia Ankara boost vaccine. Serum and liver samples were collected during vaccination and subsequently longitudinally to analyze HBV-specific immune responses and viral parameters.

**Results:**

HBV antigen levels before vaccination did not significantly influence the hepatitis B surface antibody (anti-HBs) response (*p* >0.9); however, they had a significant effect on the vaccine-induced CD8 T-cell response. *TherVacB* induced activated core-specific CD8^+^ T cells that efficiently infiltrated the liver in low- and high-titer HBV carriers. Low-titer HBV carrier mice achieved sustained viral clearance and memory T-cell formation, whereas high-titer mice exhibited only transient viral reduction, followed by CD8^+^ T-cell dysfunction and failure to clear the infection. The persistence of HBV infection and constant exposure to high antigen levels led to the overexpression of exhaustion markers, such as PD-1 and TOX (*p* <0.01), as well as the co-stimulatory receptor 4-1BB (*p* <0.01). Core-specific CD8^+^ T cells remained trapped in the liver, progressively lost their effector function, and were eventually eliminated.

**Conclusions:**

These findings identify critical time points that influence vaccine-induced CD8 T-cell responses, suggesting that early intervention targeting T-cell dysfunction may enhance therapeutic vaccination outcomes in chronic HBV infection.

**Impact and implications:**

This study demonstrates that baseline HBV antigen load critically determines the durability of vaccine-induced CD8^+^ T-cell immunity. Although therapeutic vaccination initially elicited liver-infiltrating, HBV-specific CD8^+^ T cells irrespective of the viral titer, only low-titer infection permitted sustained viral clearance and memory T-cell formation. In high-titer HBV carriers, persistent antigen exposure drove progressive T-cell exhaustion and deletion, resulting in transient viral control and treatment failure. Importantly, the data identify a critical time window in which early intervention to prevent or reverse T-cell dysfunction may be decisive. Clinically, these findings support combining therapeutic vaccination with antigen-lowering or immunomodulatory strategies applied early to achieve durable immune control in chronic HBV infection.

## Highlights

• Baseline HBV antigen load determines durability of vaccine-induced CD8^+^ T cells.
• Therapeutic vaccination induces CD8^+^ T-cell infiltration into the liver regardless of titer.
• High antigen load drives CD8^+^ T-cell dysfunction, deletion, and treatment failure.
• Early immune modulation during the therapeutic vaccination may enhance durable HBV control.

## Graphical abstract

**Figure:**
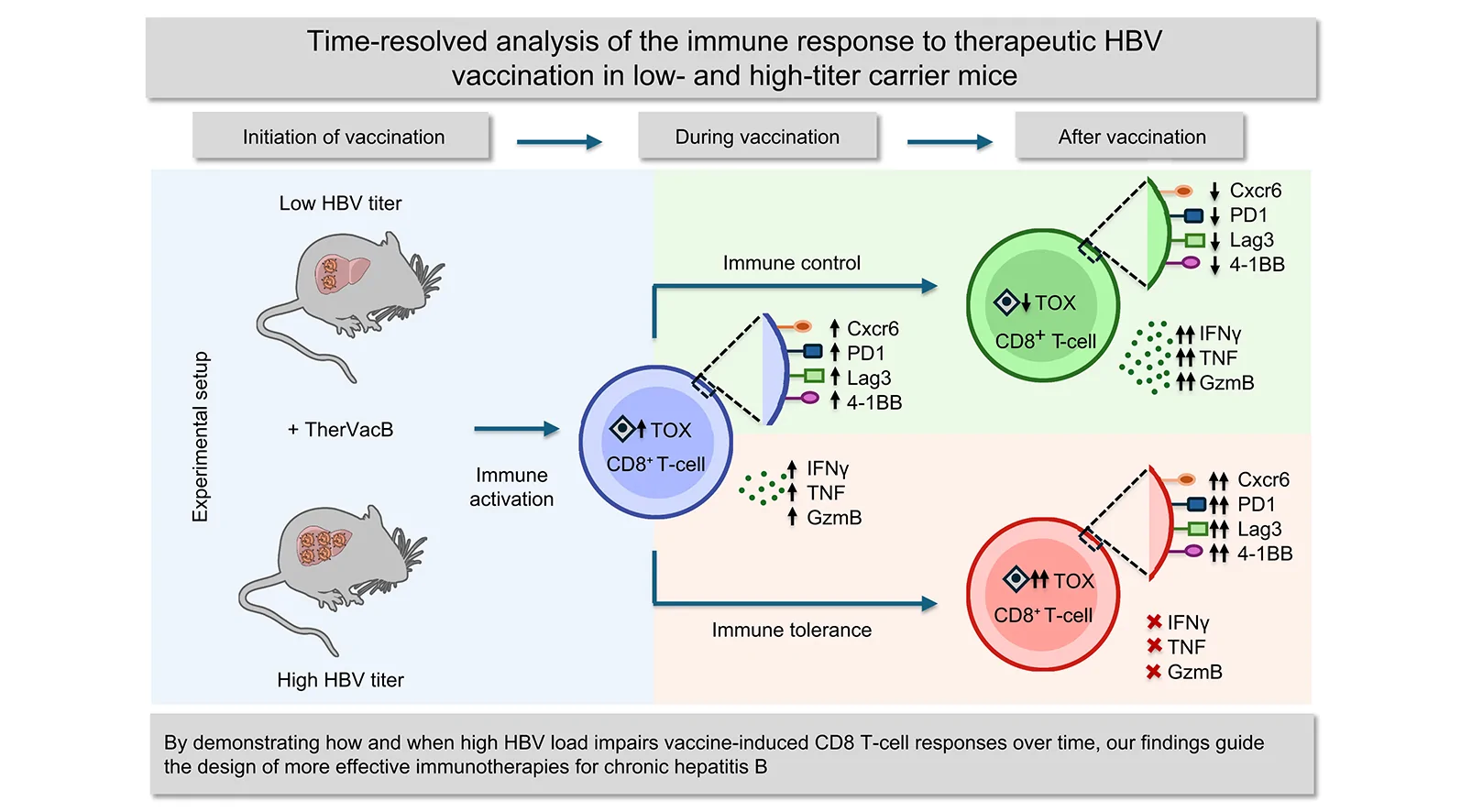

## Introduction

Chronic HBV infection affects 254 million people worldwide. It is associated with a high risk of developing HBV-associated liver cirrhosis and hepatocellular carcinoma, leading to 1.1 million deaths annually.^1^ Currently available treatments for chronic hepatitis B with pegylated-interferon-α or nucleos(t)ide analogs are either inapplicable for many patients or rarely achieve HBV cure.[2], [3]

Chronic HBV infection is associated with inadequate activation of both innate and adaptive immunity, resulting in a scarcity and dysfunction of virus-specific CD8^+^ T cells that are unable to eliminate infected hepatocytes and clear the infection.[4], [5] In contrast, acute HBV infection—and, in sporadic cases, chronic HBV infection—is resolved by effective, multi-specific virus-specific CD8 T-cell responses.^6^ Therefore, activating the patient’s immune system with a potent therapeutic vaccine to control chronic HBV infection is a promising strategy for achieving a cure for HBV.^5^

We developed a therapeutic hepatitis B vaccine, *TherVacB*, which follows a heterologous protein prime/recombinant modified vaccinia Ankara (MVA) boost scheme^7^ that is currently under evaluation in clinical trials. For prime immunization, recombinant HBV S and core antigens, which form virus-like particles (VLPs), are used to induce neutralizing antibodies that capture circulating HBsAg, prevent virus spread, and prime HBV-specific CD4^+^ and CD8^+^ T cells. The subsequent boost with recombinant MVA, designed to target 95% of circulating HBV isolates, aims to expand virus-specific CD8 T-cell responses.[8], [9], [10] In preclinical mouse models with low to intermediate HBV levels, *TherVacB* elicited strong HBV-specific immune responses and long-term immune control of HBV.[8], [11] However, high levels of HBV antigens impaired vaccine-induced HBV-specific CD8 T-cell response and antiviral efficacy. Reducing HBV antigen levels using siRNA targeting HBV improved the vaccine efficacy, indicating that low antigen levels are required for effective CD8 T-cell response and viral clearance.[12], [13] Subsequent studies have demonstrated that the level of antigen expressed in the liver, rather than circulating antigen levels, is decisive for T-cell function.[14], [15] Nevertheless, in clinical studies, siRNA-mediated HBV reduction alone had only limited effects on restoring HBV-specific immunity,^16^ highlighting that additional mechanisms contribute to vaccine unresponsiveness in high-titer HBV carriers. This remains to be fully elucidated.

In this study, we longitudinally compared antiviral immune responses induced by therapeutic vaccination in low- and high-titer HBV carrier mice across the consecutive stages of immunization, with a particular focus on liver-associated virus-specific CD8 T-cell responses. We confirmed that persistent exposure to high antigen levels is a decisive determinant of vaccine efficacy. Our immunologic and viral analyses revealed the kinetics of HBV-specific CD8^+^ T-cell expansion, functional loss, and upregulation of inhibitory markers in high-titer infection. By time-resolved analysis of CD8^+^ T-cell impairment, we identified a critical window for therapeutic intervention that could enhance the efficacy of therapeutic vaccination in high-titer carriers.

## Material and methods

### Ethical statements and animal experiments

Animal experiments were conducted in accordance with the German regulations of the Society for Laboratory Animal Science (GV-SOLAS) and the European Health Law of the Federation of Laboratory Animal Science Associations (FELASA) and were reported according to the ARRIVE (Animal Research: Reporting of *in vivo* Experiments) guidelines. Experiments were approved by the District Government of Upper Bavaria (permission number: ROB-55.2-2532.Vet_02-18-24). Mice were housed under biosafety level 2 and specific pathogen-free conditions, in accordance with institutional guidelines.

Eight-to ten-week-old male C57BL/6J mice were purchased from Janvier Labs (Le Genest-Saint-Isle, France) and acclimatized for at least 7 days. To establish persistent low- and high-titer persistent HBV replication, mice were intravenously injected with 2 × 10^9^ or 1 × 10^10^ genome equivalents of the adeno-associated-virus (AAV)-HBV1.2 vector encoding a 1.2-fold over-length HBV genome of genotype D (AAV-HBV) 6 weeks before vaccination. Low-titer mice were defined by an HBsAg level of 133.9 (±69.7) IU/ml, whereas high-titer mice were defined by an HBsAg level of 1093.8 (±665.2) IU/ml at baseline. The animals’ overall well-being and weight were monitored weekly. The group size (n ≥4) was determined by a statistician to ensure adequate power for reliable statistical analyses.

### Heterologous protein prime/MVA boost vaccination regimen

Mice received the therapeutic heterologous protein prime/MVA boost vaccine (*TherVacB*) as previously described.^7^ In brief, each mouse was intramuscularly injected into both hind limbs with a dosage of 10 μg each of VLPs consisting of HBV S (genotype A; Biovac, Cape Town, South Africa) and core (genotype D; APP Lativijas Biomedicinas, Riga, Latvia) proteins formulated with 10 μg of c-di-AMP as an adjuvant (InvivoGen, San Diego, CA, USA) at Weeks 0 and 2. A booster immunization was administered at Week 4, with each mouse receiving an intramuscular injection of 3 × 10^7^ infectious units of recombinant MVA expressing the corresponding HBV S and core proteins (MVA-S and MVA-core, respectively).^17^

### Isolation of splenocytes and liver-associated lymphocytes and flow cytometry analysis

Single-cell suspensions of murine splenocytes and liver-associated lymphocytes (LALs) were isolated as previously described.[7], [12]

HBV-specific CD8^+^ T cells were detected through staining with MHC class I multimers conjugated with the H-2^Kb^-restricted HBV-specific S_190–197_ (S_190_; VWLSAIWM) or core_93–100_ (C_93_; MGLKFRQL). MVA-derived multimer B8R_20–27_ (MVA_B8R_; TSYKFESV) was used as a control. Multimers were prepared as previously described.^18^ Before use, multimers were labeled with streptavidin-APC (Thermo Fisher Scientific, Waltham, MA, USA) as previously described.^12^ Cell surface staining was performed using anti-CD4, anti-CD8, anti-LAG3, anti-PD1, anti-4-1BB, anti-CXCR6, and anti-CD44 antibodies (Table S1). The lymphocyte populations were characterized using anti-CD45.2, anti-CD3, anti-CD19, anti-CD4, anti-CD8, anti-NK1.1, and anti-CD25 antibodies (Table S1). Dead cells were excluded from analysis by staining with Fixable Viability Dye eF780 (Thermo Fisher Scientific). For intranuclear staining, cells were washed and fixed using the FOXP3 Fixation/Permeabilization kit (Thermo Fisher Scientific) according to the manufacturer’s instructions. Cells were then permeabilized for 4 h and stained intranuclearly with anti-TOX or anti-FOXP3 antibodies.

For intracellular cytokine staining (ICS), splenocytes and LALs were stimulated overnight with HBsAg-derived (Table S2) and HBcAg-derived (Table S3) overlapping peptide pools^9^ or with the control OVA_S8L_ peptide (SIINFEKL). Cell surface staining was performed using anti-CD4 and anti-CD8 antibodies (Table S1). interferon γ (IFNγ), tumor necrosis factor (TNF), and granzyme B (GzmB) were detected intracellularly as previously described.^7^

Absolute cell numbers were determined using CountBright™ counting beads (Thermo Fisher Scientific) according to the manufacturer’s instructions. Data were acquired using a CytoFlexS flow cytometer (Beckman Coulter, Indianapolis, IN, USA) and analyzed using FlowJo software v10.8 (Tree Star, Woodburn, OR, USA).

### Serological and viral analyses

Serum HBsAg, HBeAg, hepatitis B e antibody (anti-HBe), and hepatitis B surface antibody (anti-HBs) levels were quantified on an Architect™️ platform (Abbott, Wiesbaden, Germany), and hepatitis B core antibody (anti-HBc) levels were measured using the LIAISON® (DiaSorin, Saluggia, Italy) as previously described.^7^ Serum alanine aminotransferase (ALT) activity was determined using the Reflotron® GPT/ALT tests (Roche Diagnostics, Mannheim, Germany) or the Respons910 ALAT/GPT kit (DiaSys, Holzheim, Germany) according to the manufacturer’s instructions.

H&E staining of deparaffinized liver sections was performed according to standard protocols. Histopathological evaluation and inflammation scoring were performed as previously described, using the Desmet grading system,^19^ which has been adapted for murine hepatitis. For immunohistochemistry, formalin-fixed liver sections were stained with an anti-HBcAg antibody (LS Bio, Newark, CA, USA; 1:50 dilution) as previously described.^13^ The number of core-positive hepatocytes was determined in 10 randomly selected fields at 20 × magnification and quantified per square millimeter (mm^2^).

Intrahepatic HBV DNA and RNA were extracted from 20 and 30 mg of liver tissue, respectively, using Nucleospin Tissue kits (Macherey-Nagel, Düren, Germany) according to the manufacturer's instructions. HBV DNA (rcDNA) was quantified using PCR with SYBR Green on a LightCycler instrument (Roche Diagnostics, Rotkreuz, Switzerland) and normalized to the Prp gene (Table S4). The 3.5-kb pregenomic RNA (pgRNA) was quantified using real-time PCR with SYBR Green and normalized to GAPDH (Table S5).

### HBV neutralization assay

HBV neutralization assay was performed as previously described.[20], [21], [22] In brief, HepG2-NTCP (in-house produced) cells were seeded at 70–80% confluency in 2.5% DMSO (Merck, Darmstadt, Germany) 3 days before infection with purified NanoLuc-expressing HBV (rHBV). Pooled mouse sera from each vaccination group were serially diluted in medium with 2.5% DMSO and 4% PEG-6000 (Merck), mixed with rHBV, and incubated for 30 min at room temperature. Cells were inoculated overnight, washed with PBS, and cultured in fresh medium with 2.5% DMSO. Supernatants were collected on day 7 after infection. Luminescence was measured using an Infinite F200 Tecan plate reader (Tecan Group, Männedorf, Switzerland) with 10 μl of supernatant and 100 μl of substrate solution (1 μM coelenterazine dissolved in acidified methanol, diluted 1:1,000 in PBS supplemented with 0.1% Tween-20; PJK, Kleinblittersdorf, Germany). Inhibition of infection was normalized to naïve serum controls, and half-maximal inhibitory concentration (IC_50_) values were determined using four-parameter nonlinear regression in GraphPad Prism (GraphPad Software Inc., San Diego, CA, USA).

### Statistical analyses

In all graphs, data are presented as mean ± SEM. Analyses were conducted using GraphPad Prism version 9.5 or 10.1 (GraphPad Software Inc.), applying one- or two-way ANOVA or the Mann-Whitney *U* test. Values of *p* ≤0.05 were considered significant, and only statistically significant differences are shown.

## Results

### High HBV titers prevent HBsAg and HBeAg seroclearance after therapeutic vaccination

To dissect differences in *TherVacB*-mediated antiviral responses between mice with low and high HBV levels, we performed a longitudinal, time-resolved analysis of HBV-related serological parameters during and after therapeutic vaccination. Eight-to ten-week-old C57BL/6J male mice were infected with different AAV-HBV doses to establish low- and high-level persistent HBV replication. Subsequently, the mice were immunized with adjuvanted core- and S-VLPs at Weeks 0 and 2 and boosted with MVA expressing HBV core and S proteins at Week 4 (Fig. 1A). Analyses were performed before initiating *TherVacB* (Week 0), during the immunizations (Weeks 2 and 3), and early (Week 5) and later (Weeks 8, 12, and 18) after completing the vaccination scheme.

**Fig. 1:**
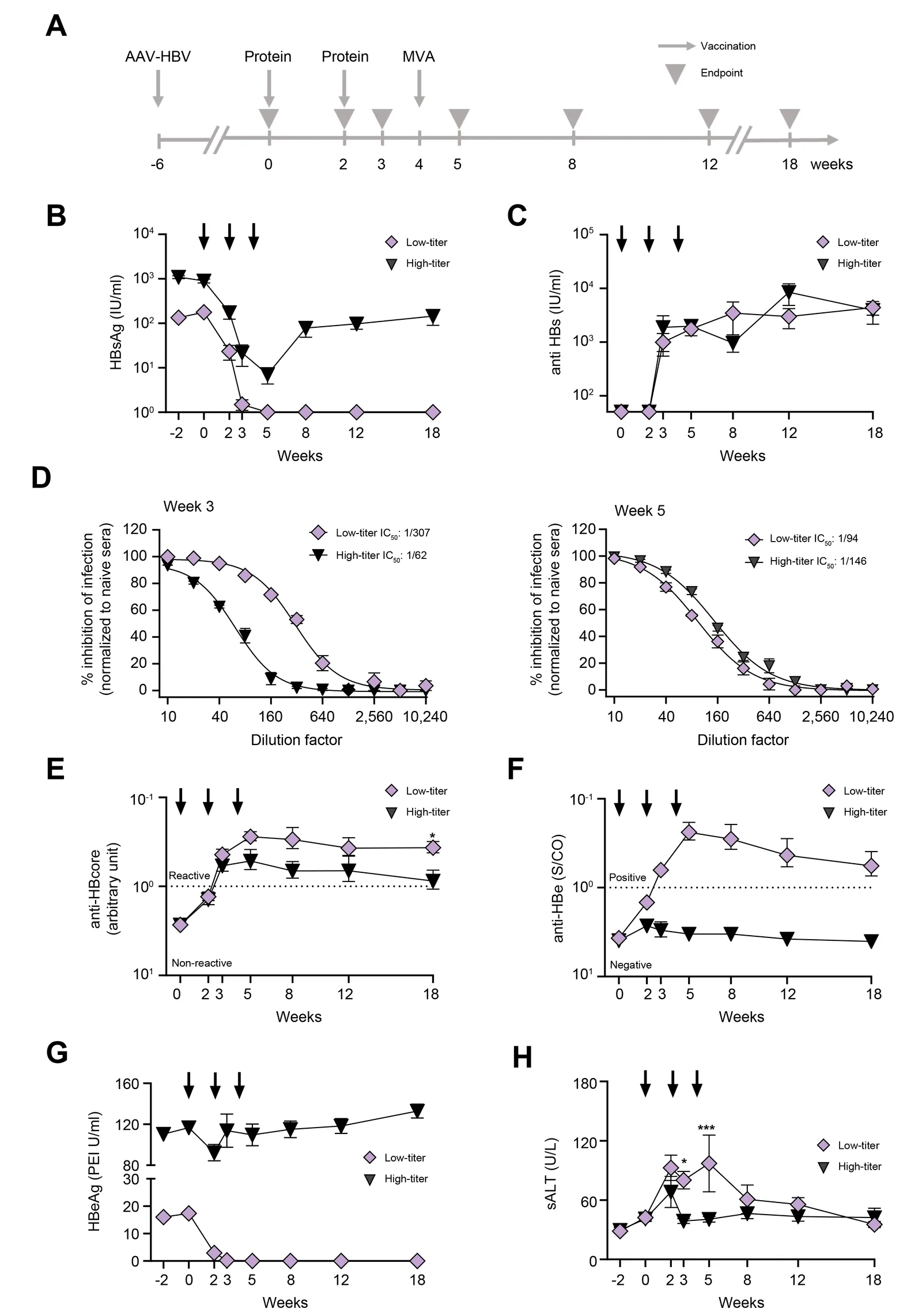
Effect of *TherVacB* on the HBV-related serological parameters. (A) C57BL/6J mice were intravenously injected with AAV-HBV1.2 6 weeks before vaccination to establish low- and high-level persistent HBV replication. Mice were then immunized intramuscularly (black arrows) following the *TherVacB* regimen, twice using a combination of adjuvanted HBV S- and core-VLPs (Weeks 0 and 2) (10 μg each) and once with MVA expressing HBV-core and –S proteins (Week 4) (3 × 10^7^ IFU each). The final analyses were performed at the indicated time points (gray triangles). Time kinetics of serum (B) HBsAg and (C) anti-HBs in low-*vs*. high-titer HBV carrier mice. (D) The percent inhibition of infection sera from low- and high-titer mice collected at Weeks 3 and 5 was determined by a neutralization assay using recombinant HBV expressing NanoLuc luciferase. IC_50_ values determined from neutralization curves are shown. Time kinetics of serum (E) anti-HBc, (F) anti-HBe, (G) HBeAg, and (H) sALT levels in low-*vs*. high-titer HBV carrier mice. Statistical analyses were performed using two-way ANOVA. Asterisks mark statistically significant differences: ∗*p* ≤0.05; ∗∗∗*p* ≤0.001. Data are presented as mean and SEM (n ≥5). AAV, adeno-associated virus; anti-HBs, hepatitis B e antibody; IFU, infectious units; MVA, modified vaccinia Ankara; sALT, serum alanine transaminase; *TherVacB*, therapeutic hepatitis B vaccine; VLP, virus-like particle.

With each vaccination, antigen levels decreased in both low- and high-titer mice, reaching their lowest values at Week 5, that is, 1 week after the MVA boost (Fig. 1B). Although in low-titer HBV carriers HBsAg levels remained below the detection limit, HBsAg levels quickly rebounded in high-titer mice, resulting in only a 1-log_10_ lower level at Week 18 than at baseline. Nevertheless, the amount of anti-HBs antibodies induced was comparable between low- and high-titer mice over the monitoring period (Fig. 1C). To further investigate whether vaccine-induced anti-HBs differ in their neutralization capacity between low- and high-titer mice, we performed a neutralization assay in HepG2-NTCP cells using reporter HBV expressing NanoLuc luciferase. Although sera from low-titer mice demonstrated approximately fivefold stronger HBV neutralization than the sera from high-titer mice at Week 3, this difference was not sustained at Week 5 (Fig. 1D) or Week 18 (Fig. S1). As with anti-HBs antibodies, anti-HBc antibodies were first detected after the second protein vaccination at Week 3 (Fig. 1C,E), indicating that at least two vaccinations are required to induce effective antibody responses. In low-titer mice only, anti-HBc levels further increased after the MVA boost (Fig. 1E).

In contrast to patients with chronic hepatitis B, serum HBeAg is not spontaneously cleared in AAV-HBV-transduced mice; HBeAg seroconversion indicates the elimination of persistent HBV infection.[7], [12] Low-titer HBV carriers seroconverted to anti-HBe already after the second protein vaccination (Fig. 1F). Consequently, HBeAg dropped below the detection limit in low-titer mice after the MVA boost (Fig. 1G), which was preceded by a transient serum alanine transaminase (sALT) flare, indicating the elimination of HBV-positive hepatocytes (Fig. 1H). In contrast, high-titer mice showed no detectable increase in ALT except at Week 2, suggesting that the short-term antiviral effect may be induced even in the presence of high HBV levels.

### High HBV titers prevent efficient elimination of the infected hepatocytes after therapeutic vaccination

To further substantiate our findings and confirm the effects of *TherVacB* on HBV infection status in low-*vs*. high-titer mice, we assessed liver inflammation and analyzed viral parameters in liver tissue collected at consecutive time points.

Although we observed immune cell infiltration into the liver parenchyma in both low- and high-titer mice following *the TherVacB* vaccination (Fig. 2A), no significant histopathological changes were detected, with only minimal or mild inflammation scores (Fig. 2B). Although low-titer mice exhibited a gradual decrease in the number of core-positive hepatocytes during vaccination, ultimately becoming negative after completion of the entire *TherVacB* scheme, the number of HBV-positive hepatocytes remained high throughout the monitoring period in high-titer mice (Fig. 2C,D). Although the clearance of HBV from the liver took longer than from the blood, *TherVacB* ultimately resulted in the clearance of HBV DNA and pgRNA in low-titer mice by Week 18 (Fig. 2E,F). Interestingly, the amount of intrahepatic HBV DNA and pgRNA decreased at Week 2 in high-titer mice but eventually rebounded to almost the starting level by Week 18 (Fig. 2E,F).

**Fig. 2:**
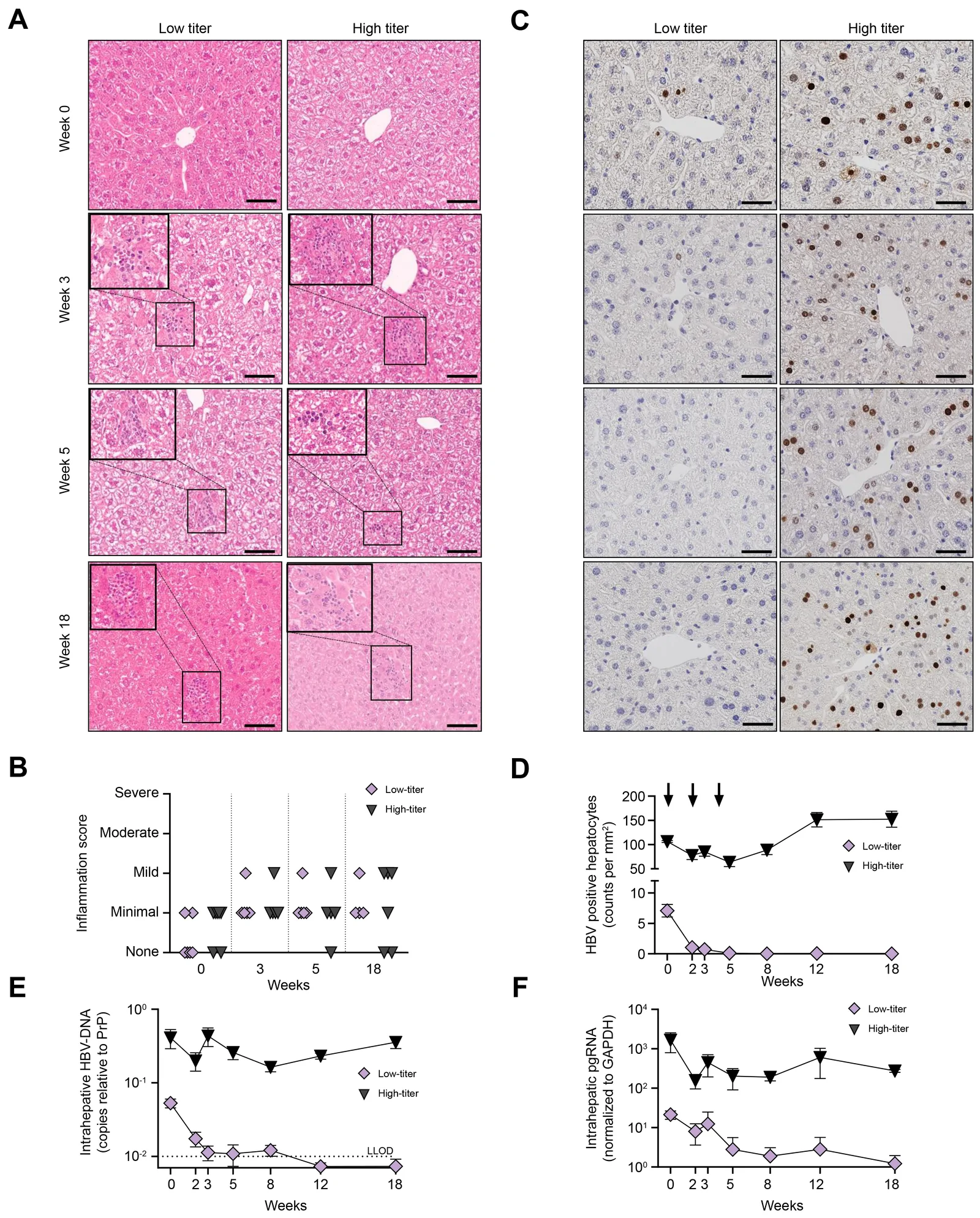
Effect of *TherVacB* on the HBV persistence in the liver. High- and low-titer HBV carriers were immunized with *TherVacB*, as shown in Fig. 1A; analyses were performed at the indicated time points. Representative images of murine liver (A) H&E staining and (B) histological scoring to assess liver inflammation in individual mice. (C) immunohistochemistry staining and (D) quantification (counts/mm^2^) of HBV core-positive hepatocytes (brown nucleus) at the endpoints. Intrahepatic (E) HBV DNA detected in murine liver tissue lysates by qPCR and normalized to the Prp and (F) pgRNA detected in liver tissue lysates by qPCR and normalized to the GAPDH. Data are presented as mean and SEM (n ≥4). pgRNA, pregenomic RNA; qPCR, quantitative PCR; *TherVacB*, therapeutic hepatitis B vaccine.

These results demonstrate that *TherVacB* rapidly eliminates HBV from the livers of low-titer carriers but fails to do so in high-titer HBV carrier mice.

### Therapeutic vaccination triggers lymphocyte infiltration into the liver

To investigate how *TherVacB* modulates the dynamics of the immune response in low-*vs*. high-titer HBV carriers in the secondary lymphoid organs, the spleen, and the site of infection, the liver, we compared counts of relevant immune cell populations during and after vaccination.

Six weeks after establishing low- and high-titer persistent HBV replication, before we applied *TherVacB* (Week 0), there were no significant differences in the counts of B cells, CD4^+^ and CD8^+^ T cells, and natural killer (NK) cells between the groups of mice, in either the spleen or the liver (Fig. 3A–D). One week after the complete *TherVacB*, at Week 5, the numbers of all examined effector cell populations increased substantially, regardless of the initial infection levels. The only differences observed were higher CD8^+^ T cell counts (Fig. 3C) and NK cell counts (Fig. 3D) in the spleen at Week 5 and B cell counts in the liver at Week 18 (Fig. 3A) in high-titer mice, whereas the counts of NK cells were significantly increased in the livers of low-titer mice at Week 5 (Fig. 3D).

**Fig. 3:**
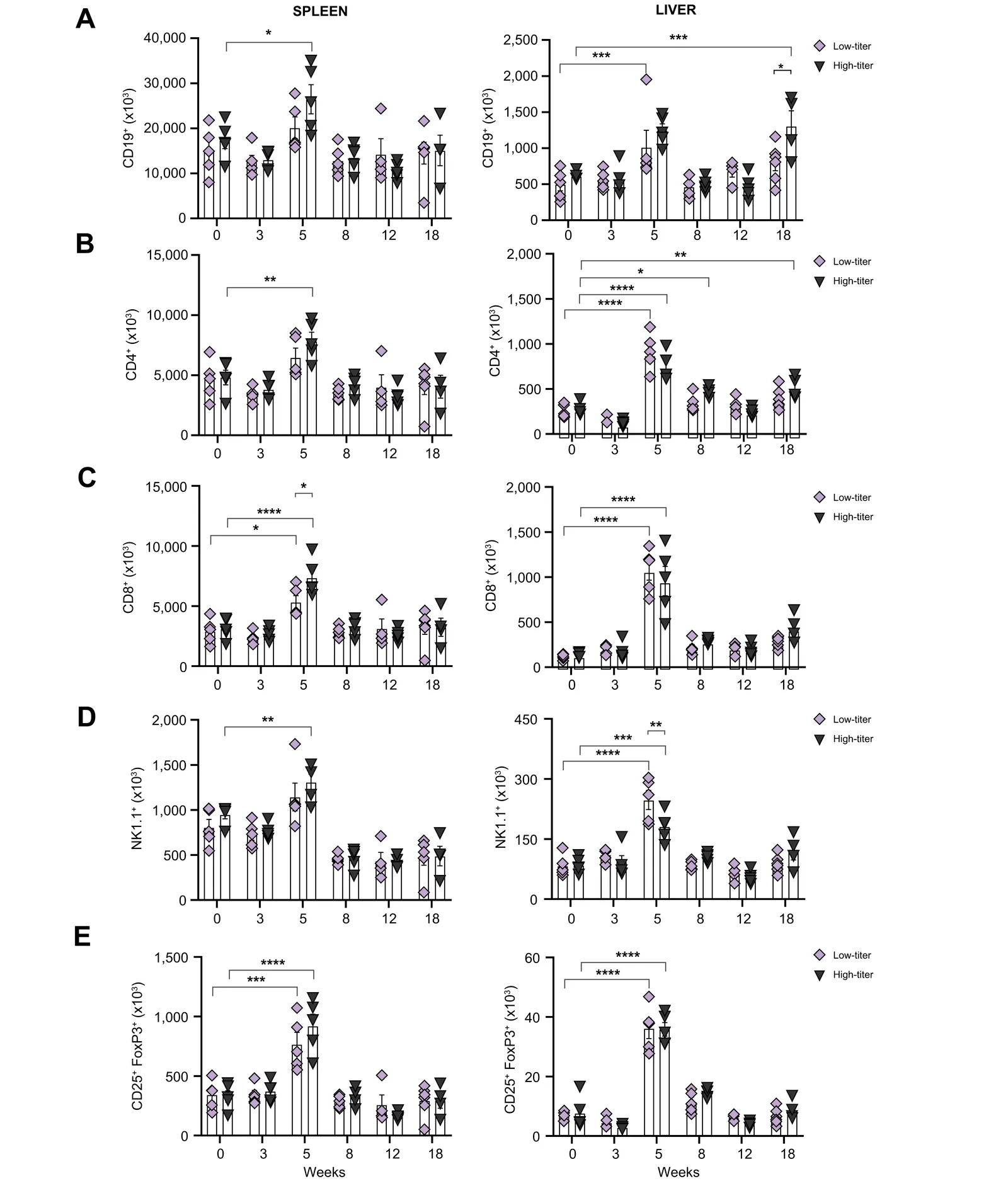
Changes in lymphocyte numbers in the spleen and liver during and after *TherVacB* vaccination. High- and low-titer HBV carriers were immunized with *TherVacB*, as shown in Fig. 1A; analyses were performed at the indicated time points. Numbers of (A) B, (B) CD4^+^ T, (C) CD8^+^ T, (D) NK, and (E) Treg cells in the spleen and the liver detected by multicolor flow cytometry. Cell counts were determined using CountBright™ absolute counting beads. Statistical analyses were conducted using one-way or two-way ANOVA, comparing groups at each independent time point and with Week 0. Asterisks mark statistically significant differences: ∗*p* ≤0.05; ∗∗*p* ≤0.01; ∗∗∗*p* ≤0.001; ∗∗∗∗*p* ≤0.0001. Data are presented as e mean and SEM (n ≥4). NK, natural killer; *TherVacB*, therapeutic hepatitis B vaccine; Treg, regulatory T cell.

Given that high-titer mice were unable to resolve persistent HBV infection after *TherVacB*, we next investigated whether regulatory T cells (Tregs) could play a role in suppressing vaccine-mediated immunity in high-titer HBV carriers. We compared the numbers of Tregs between low- and high-titer carriers but found no difference in the counts of splenic or intrahepatic Tregs at any of the time points examined (Fig. 3E).

These data demonstrate that *TherVacB* resulted in rapid expansion of the major immune cell populations in the spleens and livers of all immunized mice, with only subtle differences in the counts of hepatic NK and B cell populations that could be detected between low- and high-titer mice. Moreover, our data suggest that Tregs are unlikely to limit the efficacy of *TherVacB* in high-titer AAV-HBV mice.

### Vaccine-induced HBV-specific CD8^+^ T cells experience different fates depending on the initial HBV levels

Given that HBV-specific CD8^+^ T cells are key to viral clearance,^23^ we examined how HBV infection levels shape vaccine-induced CD8^+^ T-cell immunity during and after vaccination. We first compared the numbers of HBV core- and S-specific CD8^+^ T cells in the livers and spleens of low- and high-titer HBV carriers, using C_93_ and S_190_ MHC class I multimers, respectively.

In the liver, counts of C_93_-specific CD8^+^ T cells steadily increased after each protein immunization in low- and high-titer HBV carriers, achieving similar numbers at Weeks 2 and 3 (Fig. 4A), indicating that *TherVacB* is capable of expanding HBV-specific CD8^+^ T cells in both settings. In low-titer HBV carriers, intrahepatic C_93_-specific CD8^+^ T cells vigorously expanded following MVA booster vaccination, reaching peak levels at Week 5. Following HBV elimination, the C_93_-specific CD8^+^ T cell population contracted but then stabilized (Fig. 4A). In high-titer carriers, by contrast, C_93_-specific CD8^+^ T cells did not further expand after MVA boost vaccination, but instead gradually declined in numbers and were barely detectable by Week 18. In low-titer carriers, C_93_-specific CD8^+^ T cells became detectable in the spleen at Week 5, correlating with the resolution of persistent HBV infection (Fig. 4B). In the spleens of high-titer carriers, no C_93_-specific CD8^+^ T cells could be detected at any time point. Counts of MVA-specific CD8^+^ T cells were comparable between low- and high-titer carriers and readily detected in spleen and liver (Fig. S2A and B), demonstrating that the observed differences in numbers of C_93_-specific CD8^+^ T cells were strictly HBV specific.

**Fig. 4:**
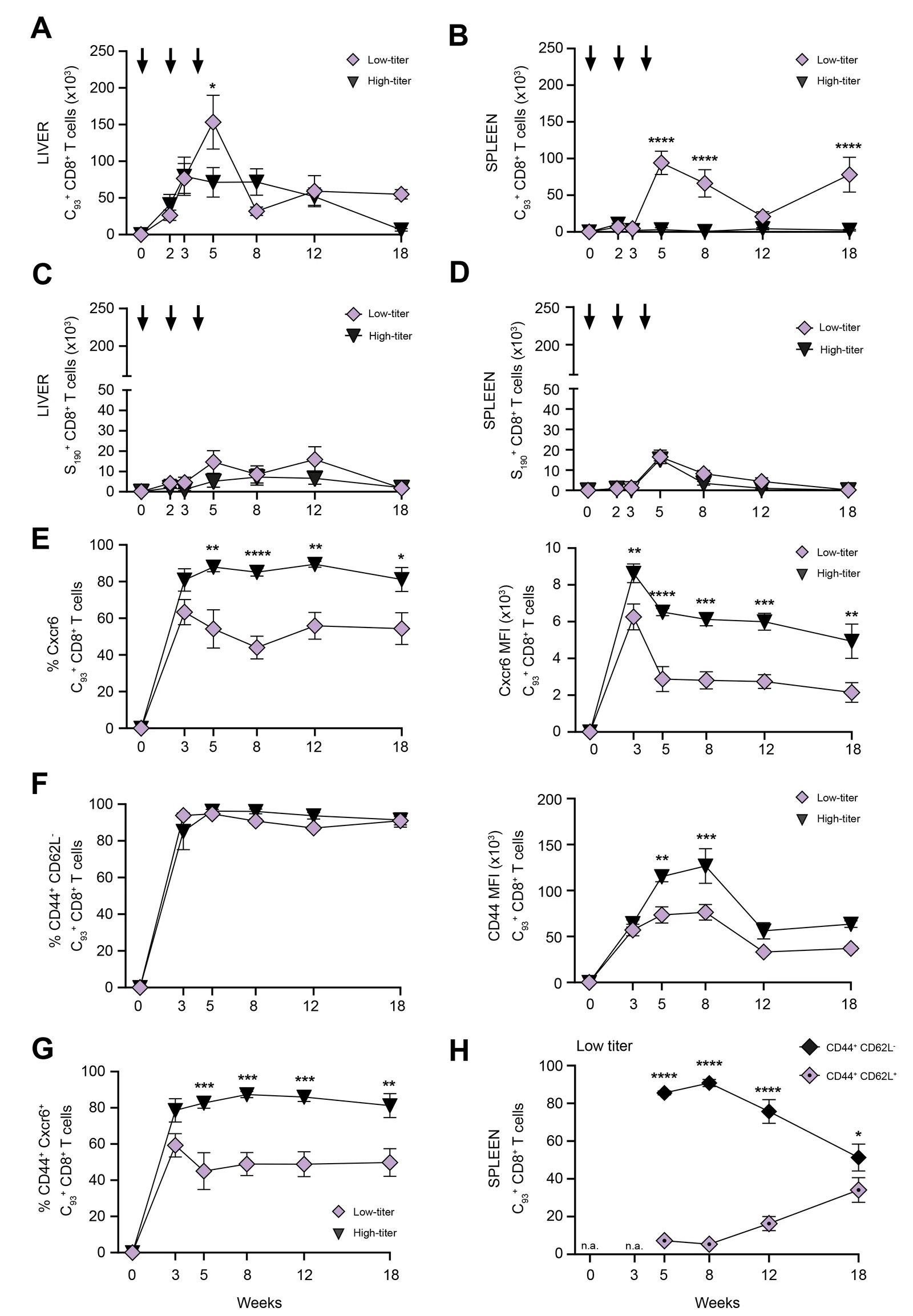
Phenotypic analysis of *TherVacB*-induced CD8^+^ T cells. High- and low-titer HBV carriers were immunized with *TherVacB*, as shown in Fig. 1A; analyses were performed at the indicated time points. Numbers of (A) liver-associated and (B) splenic core-specific and (C) liver-associated and (D) splenic S-specific CD8 T cells in low- and high-titer mice detected by staining with C_93_ and S_190_ multimers *ex vivo*. Cell counts were determined using CountBright™ absolute counting beads. Frequencies of C_93_-specific liver-associated (E) Cxcr6^+^ and (F) CD44^+^ CD62L^-^ CD8^+^ T cells and their MFIs. (G) Frequencies of C_93_-specific liver-associated CD44^+^Cxcr6^+^ CD8^+^ T cells. (H) Frequencies of splenic C_93_-specific effector and effector memory (CD44^+^CD62L^-^) and central memory (CD44^+^CD62L^+^) CD8^+^ T cells in low-titer mice. Statistical analyses were performed using two-way ANOVA. Asterisks mark statistically significant differences: ∗*p* ≤0.05; ∗∗*p* ≤0.01; ∗∗∗*p* ≤0.001; ∗∗∗∗*p* ≤0.0001. Data are presented as mean and SEM (n ≥4). MFI, mean fluorescent index; n.a., not applicable; TherVacB, therapeutic hepatitis B vaccine.

Surprisingly, S_190_-specific CD8 T cells were detected only after a complete vaccination scheme at Week 5 in both low- and high-titer HBV carrier mice (Fig. 4C,D). The number of S_190_-specific CD8^+^ T cells in the livers and spleens of low- and high-titer carriers remained significantly lower than that of C_93_-specific cells and, interestingly, showed only minor differences between both groups.

We next investigated how different levels of pre-existing HBV replication influence the activation and memory formation of liver-infiltrating vaccine-elicited HBV-specific CD8^+^ T cells. Because the number of S_190_-specific CD8^+^ T cells in both groups of mice was too low to allow a reliable phenotypic analysis, we focused instead on C_93_-specific CD8^+^ T cells associated with the liver. We analyzed the expression of Cxcr6, a chemokine receptor that helps guide CD8^+^ T cells to the liver,[24], [25] and found significantly more Cxcr6^+^ T cells in livers with high HBV antigen expression (Fig. 4E). Interestingly, not only the fraction of Cxcr6-positive C_93_-specific CD8^+^ T cells but also the expression levels of Cxcr6 per cell (mean fluorescent index [MFI]) were significantly higher in high-titer than in low-titer HBV carriers (Fig. 4E). In contrast, we found a very low frequency of Cxcr6^+^ MVA-specific CD8^+^ T cells (Fig. S2C).

We found that almost all *TherVacB*-induced C_93_-specific CD8^+^ T cells in the liver showed an effector/effector memory phenotype (CD44^+^CD62L^-^) in both groups of mice. The expression of activation marker CD44 on C_93_-specific CD8^+^ T cells isolated from high-titer carriers increased at Weeks 5 and 8 (Fig. 4F and Fig. S2D). Most of the C_93_^+^ CD8^+^ T cells co-expressed Cxcr6 and CD44, particularly in high-titer HBV carrier mice, suggesting an enrichment of activated liver-resident CD8^+^ T cells upon vaccination (Fig. 4G). After clearance of HBV in low-titer carriers, almost all C_93_-specific CD8^+^ T cells detected in the spleen initially showed an effector-memory phenotype, but the proportion of central-memory T cells steadily increased (Fig. 4H).

Taken together, these results demonstrate that *TherVacB* initially induced comparable numbers of HBV-specific effector CD8^+^ T cells in low- and high-titer HBV carrier mice. The MVA boost only increased the numbers of intrahepatic C_93_-specific CD8^+^ T cells in low- but not in high-titer HBV carriers. Liver-associated C_93_-specific CD8^+^ T cells showed an effector phenotype and expressed the liver-residency marker Cxcr6. In the presence of high HBV antigens, these cells remained in the liver and were gradually eliminated over time, whereas those in low-titer carrier mice established a memory phenotype.

### Vaccine-induced HBV-specific CD8^+^ T cells from high-titer HBV carriers lose their effector functions over time

Although *TherVacB* efficiently activated substantial numbers of liver-associated HBV-specific CD8^+^ T cells in high-titer carriers, it still failed to eliminate persistent HBV infection. Thus, we examined how the effector functions of *TherVacB-*induced HBV-specific CD8^+^ T cells were modulated by low and high HBV levels over time. To dissect the critical time points influencing HBV clearance, we analyzed HBV core- and S-specific IFNγ, TNF, and GzmB production using ICS.

Shortly after the first and second protein immunizations, at Weeks 2 and 3, we found low but comparable numbers of core-specific IFNγ^+^, TNF^+^, and GzmB^+^ CD8^+^ T cells in the livers of both low- and high-titer HBV carrier mice (Fig. 5A). At Week 5, following booster vaccination, the numbers of IFNγ^+^ CD8^+^ T cells reached their highest values in the low-titer mice, whereas in the high-titer mice, the numbers of functional core-specific CD8^+^ T cells decreased and became undetectable at subsequent time points. In low-titer HBV carriers, a strong and multifunctional core-specific CD8 T-cell response was detected in the spleens after Week 5, characterized by high numbers of IFNγ^+^, TNF^+^, and GzmB^+^ CD8^+^ T cells (Fig. 5B).

**Fig. 5:**
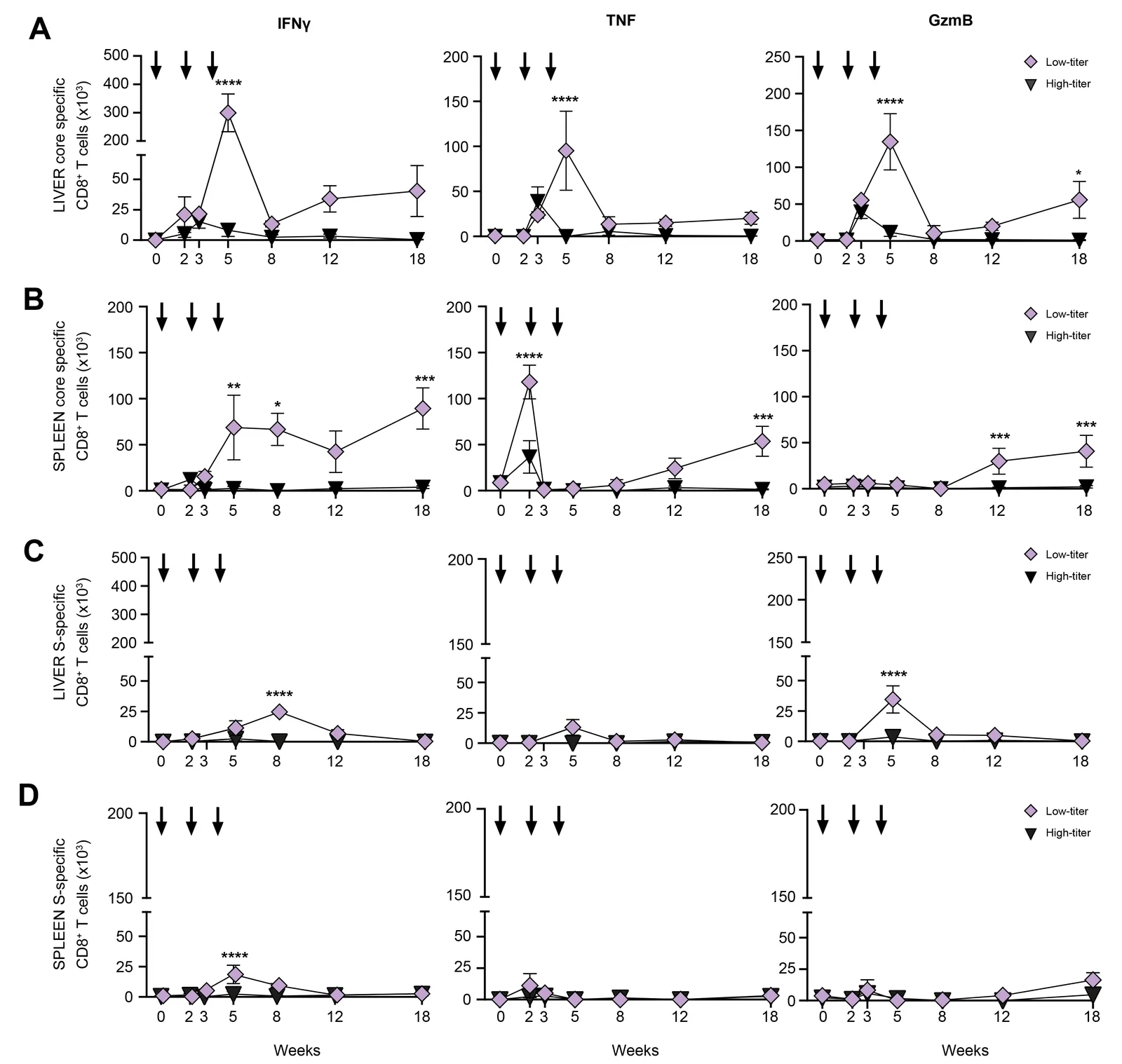
Effect of *TherVacB* on the functionality of HBV-specific CD8^+^ T cells. High- and low-titer HBV carriers were immunized with *TherVacB*, as shown in Fig. 1A; analyses were performed at the indicated time points. Numbers of (A) liver-associated and (B) splenic core-specific IFNγ^+^, TNF^+^, and GzmB^+^ CD8^+^ T cells; (C) liver-associated and (D) splenic S-specific IFNγ^+^, TNF^+^, and GzmB^+^ CD8^+^ T cells detected by intracellular cytokine staining after 16-h stimulation with corresponding HBV S- or core-derived peptide pools. Statistical analyses were performed using two-way ANOVA. Asterisks mark statistically significant differences: ∗*p* ≤0.05; ∗∗*p* ≤0.01; ∗∗∗*p* ≤0.001; ∗∗∗∗*p* ≤0.0001. Data are presented as mean and SEM own (n ≥4). GzmB, granzyme B; IFNγ, interferon γ; *TherVacB*, therapeutic hepatitis B vaccine; TNF, tumor necrosis factor.

Low-titer mice exhibited a transient effector S-specific CD8 T-cell response, albeit at a significantly lower level than the core-specific response, with IFNγ^+^ cells detected in the liver and spleen at Weeks 5 and 8, whereas GzmB^+^ cells were found only in the liver at Week 5 (Fig. 5C,D). Functional *TherVacB*-induced S-specific CD8^+^ T cells could not be detected in the livers or spleens of high-titer carrier mice at all.

Taken together, these results indicate that vaccine-elicited CD8^+^ T cells were initially functional in the livers of high-titer mice but lost their ability to produce antiviral cytokines after the MVA boost. In contrast, functional CD8^+^ T cells were readily detected in the livers and spleens of low-titer HBV carriers that cleared HBV after vaccination. The detection of only low S-specific CD8 T-cell responses, even in low-titer carriers, suggested a primary role for core-specific CD8^+^ T cells in resolving the infection.

### *TherVacB*-induced HBV-specific CD8^+^ T cells exhibit differences in the expression of immune checkpoint markers

We hypothesized that the subsequent loss of effector functions of C_93_-specific CD8^+^ T cells in the livers of high-titer carriers may be attributed to either T-cell exhaustion or the upregulation of markers reported to mediate T-cell dysfunction in response to antigen stimulation in the liver. We analyzed liver-associated C_93_-specific CD8^+^ T cells for their expression of the exhaustion-related transcription factor TOX, immune checkpoint receptors PD-1 and LAG-3,[26], [27] and the co-stimulatory molecule 4-1BB, which has recently been reported to modulate HBV-specific CD8 T-cell functions in the liver.[28], [29]

At Week 3, after protein prime vaccination, there was no significant difference in the expression levels of PD-1, LAG-3, and TOX (Fig. 6A) on C_93_-specific CD8^+^ T cells or in the frequencies of cells expressing PD-1 or co-expressing PD-1 and LAG-3 or TOX between low- and high-titer HBV carriers (Fig. 6B). Starting at Week 5 after the MVA boost, however, the expression of PD-1, LAG-3, and TOX on C_93_-specific CD8^+^ T cells increased considerably in high-titer carriers, correlating temporally with their loss of cytokine production. Moreover, the fraction of C_93_-specific CD8^+^ T cells positive for one or more of these molecules remained significantly elevated in high-titer carriers, whereas their frequencies gradually decreased in the livers of low-titer mice and were barely detectable on liver-infiltrating MVA-specific CD8^+^ T cells (Fig. S3). Interestingly, we found that the co-stimulatory molecule 4-1BB was also significantly upregulated on C_93_-specific CD8^+^ T cells in high-titer carriers following the complete vaccination scheme, with kinetics resembling those observed for the co-inhibitory markers (Fig. 6C). Moreover, most 4-1BB^+^ C_93_^+^ CD8^+^ T cells co-expressed PD-1 (Fig. 6D).

**Fig. 6:**
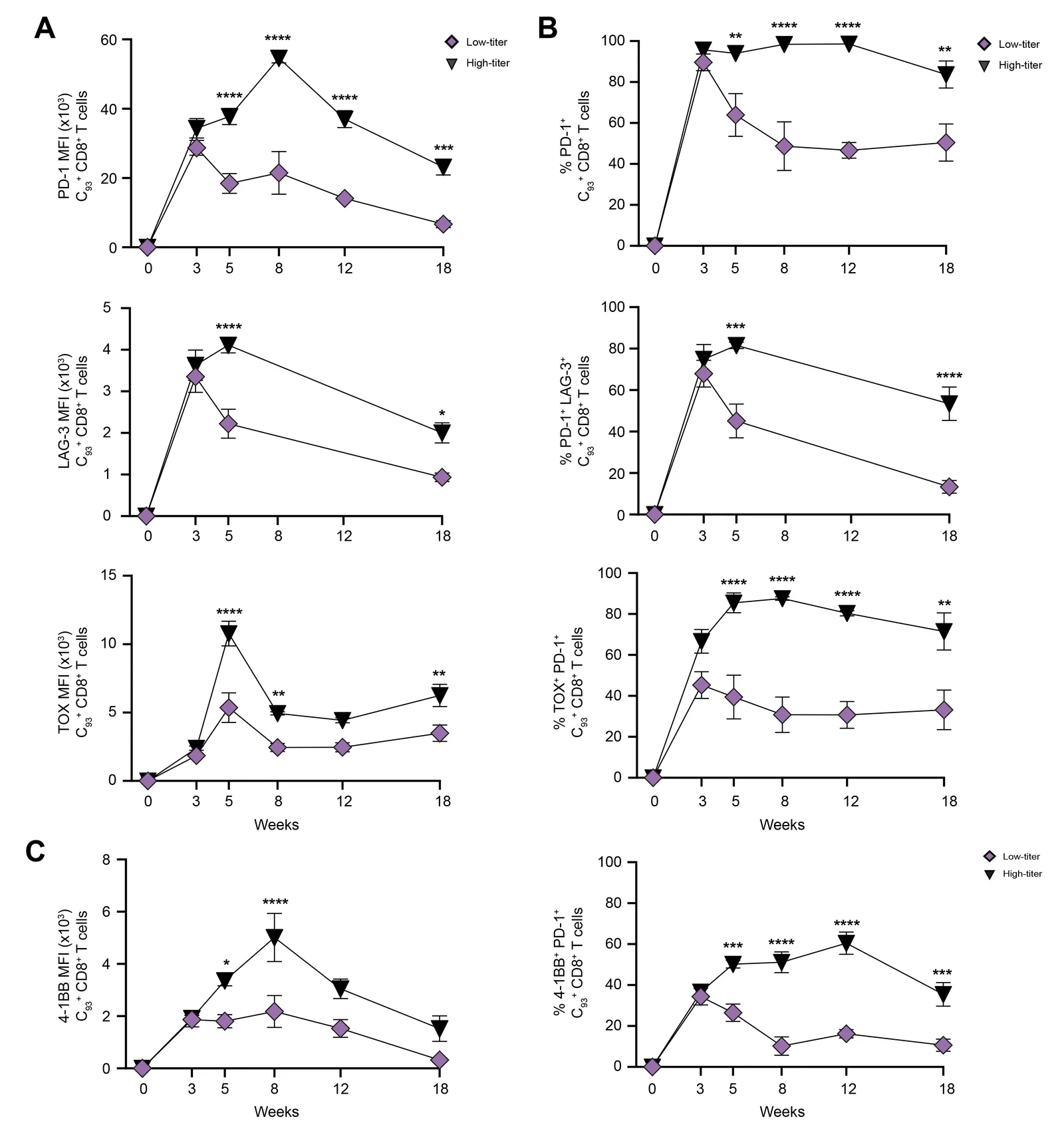
Expression of immune checkpoint markers on *TherVacB*-induced CD8^+^ T cells. High- and low-titer HBV carriers were immunized with *TherVacB*, as shown in Fig. 1A; analyses were performed at the indicated time points. (A) MFIs of PD1, Lag3, and TOX and (B) frequencies of C_93_-specific liver-associated PD-1^+^, PD-1^+^TOX^+^, and PD-1^+^LAG-3^+^ CD8^+^ T cells. (C) MFI of 4-1BB and frequencies of C_93_-specific liver-associated 4-1BB^+^ PD1^+^ CD8^+^ T cells. Statistical analyses were performed using two-way ANOVA. Asterisks mark statistically significant differences: ∗*p* ≤0.05; ∗∗*p* ≤0.01; ∗∗∗*p* ≤0.001; ∗∗∗∗*p* ≤0.0001. Data are presented as mean and SEM (n ≥4). MFI, mean fluorescent index; *TherVacB*, therapeutic hepatitis B vaccine.

Taken together, our results indicate that vaccine-induced CD8^+^ T cells exposed to high levels of HBV antigens in the liver overexpress markers associated with T-cell dysfunction, thereby suppressing their antiviral function and preventing them from eliminating HBV-infected hepatocytes.

## Discussion

High HBV antigen levels remain the major obstacle to successful therapeutic vaccination, as they suppress vaccine-elicited CD8 T-cell responses, thereby limiting the antiviral effect. For the first time, we demonstrate that immunization with our clinical candidate therapeutic vaccine, *TherVacB*, could induce HBV-specific CD8^+^ T cells in the presence of high HBV loads as efficiently as in low-level persistent infection. Although vaccine-elicited CD8^+^ T cells in high-titer HBV carriers initially displayed some antiviral efficacy, they remained trapped in the liver, progressively became dysfunctional, and were ultimately eliminated. Loss of CD8 T-cell effector functions in high-titer HBV carriers correlated with overexpression of checkpoint inhibitors, such as PD-1, exhaustion-related transcription factor TOX, and co-stimulatory molecule 4-1BB. This comprehensive and longitudinal study clearly defines a period during which vaccine-induced HBV-specific CD8 T cells lose effector function, potentially informing the timing of additional immunostimulatory therapies.

HBV-specific CD8^+^ T cells constitute a functionally and phenotypically heterogeneous population that varies with antigen specificity.^30^ We found that the *TherVacB*-elicited core-specific CD8 T-cell response displayed dynamic changes over time in quantity, function, and signature, which significantly differed between low- and high-titer HBV carrier mice and closely correlated with the antiviral effect of the vaccination. By contrast, the S-specific CD8 T-cell response remained much less prominent than the core-specific CD8 T-cell response and, surprisingly, did not depend on the initial HBV titer. This implies that core-specific CD8^+^ T cells play a key role in clearing persistent HBV infection following *TherVacB* in our mouse model. It has been previously demonstrated that the HBV S protein is less immunogenic and less accessible to CD8^+^ T cells than the core protein.^14^ The disparity between S- and core-specific CD8 T-cell responses observed in this study may also be a result of increased presentation of immunodominant core-derived epitope in the livers of C57BL/6J mice replicating HBV of genotype D. Chronic exposure to the antigen can lead to the functional impairment or deletion of antigen-specific CD8^+^ T cells.^31^ Our data demonstrate that the presence of high HBV antigen does not prevent the induction of virus-specific CD8^+^ T cells or inhibit their antiviral functions during protein immunizations with *TherVacB*. The booster vaccination with MVA, specifically designed to stimulate and expand HBV-specific CD8 T-cell responses,[9], [10] was a pivotal moment determining the outcomes of the therapeutic vaccination. In low-titer carriers, the MVA boost led to rapid expansion of liver-associated core-specific effector CD8^+^ T cells, which correlated with the elimination of persistent HBV infection. Interestingly, these highly functional Cxcr6^+^ CD8^+^ T cells detected in the livers of low-titer mice during and after HBV clearance resembled, in their characteristics, a distinct population of protective liver-resident memory (T_RM_) CD8^+^ T cells, which were also described in patients with immune control of chronic hepatitis B.^32^ Although the MVA boost resulted in strong upregulation of Cxcr6 on vaccine-induced CD8^+^ T cells in high-titer carriers, these cells failed to control HBV infection and completely lost their effector functions. In addition, without efficient elimination of HBV-positive hepatocytes, a rebound in circulating HBsAg levels was observed in high-titer mice despite the presence of anti-HBs antibodies, which were of comparable quantity and neutralization capacity to those detected in low-titer mice. Simultaneous detection of HBsAg and neutralizing anti-HBs antibodies was previously reported in patients immunized with a therapeutic vaccine containing Pre-S1, Pre-S2, and S antigens but lacking a HBV-specific CD8^+^ T-cell response.^16^ This underlines the role of vaccine-induced cytotoxic CD8^+^ T cells in reducing HBsAg reservoir in the liver and sustaining functional HBV cure.

Our previous studies have clearly shown that the antiviral effect of therapeutic vaccination depends strictly on the amount of antigen presented in the liver.[7], [12], [13] We assume that increased antigen recognition in the livers of high-titer mice provided additional stimulatory signals, which in turn ‘shut down’ the antiviral immune response. Moreover, a strong elevation of Cxcr6 on core-specific CD8^+^ T cells in the livers of high-titer carriers, as compared with low-titer carriers, could act as a ‘trap’ for activated CD8^+^ T cells, preventing their homing to the spleens. Instead, vaccine-elicited cells progressively perished in the liver of high-titer carriers, resembling the scenario of patients with chronic hepatitis B who demonstrate only very scarce S-specific CD8^+^ T cells after long-term exposure to high levels of HBsAg.^33^

There is increasing evidence that high HBV antigen load is a key determinant limiting the efficacy of therapeutic vaccination, not only in mouse models[12], [34], [35] but also in patients with chronic hepatitis B.^36^ Chronic HBV carriers, who display HBsAg levels over 1,000 IU/ml, would most likely require a combination of therapeutic vaccination with antiviral drugs or immunomodulators to achieve HBV control. We previously reported that a combination of specific siRNAs and *TherVacB* could induce a sustained antiviral effect in high-titer mice, owing to enhanced functionality of vaccine-induced CD8^+^ T cells and low expression of checkpoint markers.^12^ More recently, high antigen burden was shown to drive rapid CD8^+^ T cell dysfunction, with increased PD-1 expression and reduced cytokine production in AAV-HBV mice.^37^ Here, we demonstrate that almost all *TherVacB*-elicited CD8^+^ T cells in high-titer carriers not only continuously upregulated PD-1 but also co-expressed high levels of TOX, a transcription factor associated with persistent antigen stimulation and the severity of T-cell dysfunction in chronic hepatitis B.^27^ TOX causes epigenetic changes to maintain the exhausted state in virus-specific CD8^+^ T cells.^38^ This epigenetic imprinting implies that once vaccine-elicited CD8^+^ T cells express TOX, they may not regain function, even with further treatments. This supports our previous findings that combinatorial treatment of *TherVacB* with siRNA targeting PD-L1 allowed for a more sustained HBV control when applied during priming rather than boosting of *TherVacB*^13^ and may also explain the low efficacy of PD-1 blockade in patients during boost vaccination.^39^ Thus, we propose that additional immune modulation during therapeutic vaccination should be applied early, before CD8 T-cell function is lost.

Recent evidence suggests that dysfunction of HBV-specific CD8^+^ T cells during chronic infection may result from several novel mechanisms that differ from classical exhaustion. Virus-specific CD8^+^ T cells can be attenuated through (i) increased TGF-β signaling;^40^ (ii) upregulation of co-stimulatory TNF receptors,^29^ particularly 4-1BB; and (iii) a liver-immune rheostat, which enhances activity of transcription factor CREM and inhibits T-cell receptor signaling.^28^ We found that significantly elevated expression of 4-1BB on liver-associated vaccine-elicited CD8^+^ T cells in high-titer carriers closely correlated in time with a loss of cytokine production. Expression of 4-1BB may not only directly influence the function of vaccine-elicited HBV-specific CD8^+^ T cells in the liver but may also suggest involvement of the immune rheostat, as 4-1BB is one of the main effector molecules regulated by CREM.^28^ This suggests that targeting 4-1BB may be a promising approach to enhance the efficacy of therapeutic vaccination in high-titer HBV infection. Moreover, most of the 4-1BB^+^ CD8^+^ T cells also co-expressed PD-1, suggesting that multiple mechanisms contribute to the dysfunction of HBV-specific CD8^+^ T cells in high-titer carriers.

In conclusion, our study elucidates how high HBV antigen load suppresses antiviral CD8 T-cell immunity after therapeutic vaccination, providing valuable insights for the design of more effective immunotherapeutic strategies against chronic hepatitis B.

## Abbreviations

AAV, adeno-associated virus; ALT, alanine aminotransferase; anti-HBc, hepatitis B core antibody; anti-HBe, hepatitis B e antibody; anti-HBs, hepatitis B surface antibody; ARRIVE, Animal Research: Reporting of *in vivo* Experiments; c-di-AMP, bis-(3′,5′)-cyclic dimeric adenosine monophosphate; CTLA4, cytotoxic T-lymphocyte associated protein 4; Cxcr6, C-X-C motif chemokine receptor 6; FELASA, Federation of Laboratory Animal Science Associations; GV-SOLAS, German regulations of the Society for Laboratory Animal Science; GzmB, granzyme B; ICS, intracellular cytokine staining; IFNγ, interferon γ; LAG-3, lymphocyte-activation gene 3; LAL, liver-associated lymphocyte; MVA, modified vaccinia Ankara; NK, natural killer; PD-1, programmed cell death 1; pgRNA, pregenomic RNA; sALT, serum alanine transaminase; *TherVacB*, therapeutic hepatitis B vaccine; TNF, tumor necrosis factor; TOX, thymocyte selection-associated high-mobility group box; Treg, regulatory T cell; VLP, virus-like particle.

## Authors’ contributions

Designed the study: EAO, UP, AK. Performed the experiments: EAO, AK, JS, MG, MT, NM, HAK, SS. Analyzed the data: EAO, AK. Provided essential reagents and assistance: CM, KS, DB, PK. Provided supervision and funding: AK, UP. Wrote and finalized the manuscript: EAO, UP, AK. Read and approved the final version of the manuscript: all authors.

## Data availability

Data supporting the findings of this study are available within the article and its supplementary materials. The raw data may be available from the corresponding author upon reasonable request.

## Financial support

This study was funded by the 10.13039/501100001659German Research Foundation (10.13039/501100001659DFG) via SFB-TRR179 (project no. 272983813 to 10.13039/501100004238UP and AK), the PoC initiative by the Helmholtz and 10.13039/501100003185Fraunhofer Associations (to 10.13039/501100004238UP), the EU 10.13039/501100007601Horizon 2020 consortium TherVacB (to 10.13039/501100004238UP), the 10.13039/100009139German Center for Infection Research (10.13039/100009139DZIF) via project TTU 05.803 ‘HBV Cure’ (to 10.13039/501100004238UP), the German Ministry for Education and Research (10.13039/501100002347BMBF) via the TherVacB PLUS project, C-NATM—Cluster for Nucleic Acid Therapeutics Munich funded in the Clusters4Future initiative (funding no. 03ZU1201BA to 10.13039/501100004238UP), and SFB-TRR338 (project no. 452881907 to 10.13039/100015870DB and 10.13039/501100004238UP). EAO and MG received a PhD scholarship from the Turkish Ministry of Education. HAK received an MD scholarship from DZIF. 10.13039/100028447JS was supported by the 10.13039/100009139DZIF Academy’s maternity leave program. The funders had no role in the study design, data collection, analysis, or interpretation, the writing of the manuscript, or the decision to publish the results.

## Conflicts of interest

UP and AK are named as inventors on a patent application (PCT/EP2017/050553) describing the therapeutic vaccination scheme for TherVacB and the vector MVA-HBVac. UP is a scientific founder, shareholder, and board member of SCG Cell Therapy and serves as an *ad hoc* advisor to Aligos, Arbutus, GSK, Roche, Sanofi, Vaccitech/Barinthos, and Vir Bio. The remaining authors declare no conflicts of interest.

Please refer to the accompanying ICMJE disclosure forms for further details.
