# Supplementary material for: High HBV antigen levels drive loss of vaccine-induced CD8+ T-cell function in carrier mice over time
Source: JHEP Rep. 2026 Sep 10;8(12):102033. doi: 10.1016/j.jhepr.2026.102033 (PMC13743956; doi:10.1016/j.jhepr.2026.102033)
Supplement: Supplementary file 1 [file mmc1.pdf]

High HBV antigen levels drive loss of vaccine-induced CD8$^+$ T-cell function in carrier mice over time

Edanur Ates Öz, Jinpeng Su, Merve Gültan, Margaret Tulessin, Nikolas Müller, Hélène Anne Kerth, Swati Singh, Carolin Mogler, Katja Steiger, Jochen M. Wettengel, Dirk H. Busch, Percy A. Knolle, Ulrike Protzer, Anna D. Kosinska

Table of contents

Fig. S1……………………………………………………………………………………2

Fig. S2……………………………………………………………………………………3

Fig. S3……………………………………………………………………………………4

Table S1…………………………………………………………………………………5

Table S2…………………………………………………………………………………6

Table S3…………………………………………………………………………………6

Table S4…………………………………………………………………………………7

Table S5…………………………………………………………………………………7

Supplementary Figures

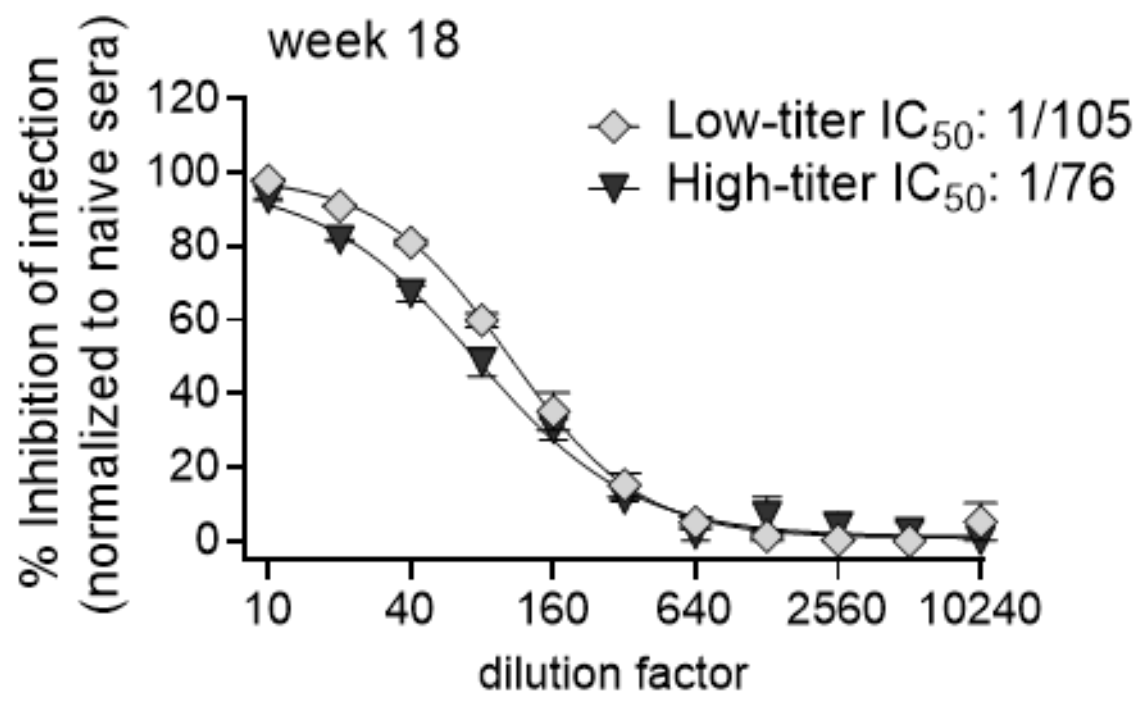

Fig. S1: Serum neutralization assay at week 18

C57BL/6J mice were intravenously injected with AAV-HBV1.2 six weeks before vaccination to establish low- and high-level persistent HBV replication. Mice were vaccinated intramuscularly following the *TherVacB* regimen. The analyses were performed at week 18, 14 weeks after completion of the vaccination scheme. The percent inhibition of infection by sera from low- and high-titer mice was determined by a neutralization assay using recombinant HBV expressing NanoLuc luciferase. IC_{50} values determined from neutralization curves are shown. The mean and SEM are shown (n = 5).

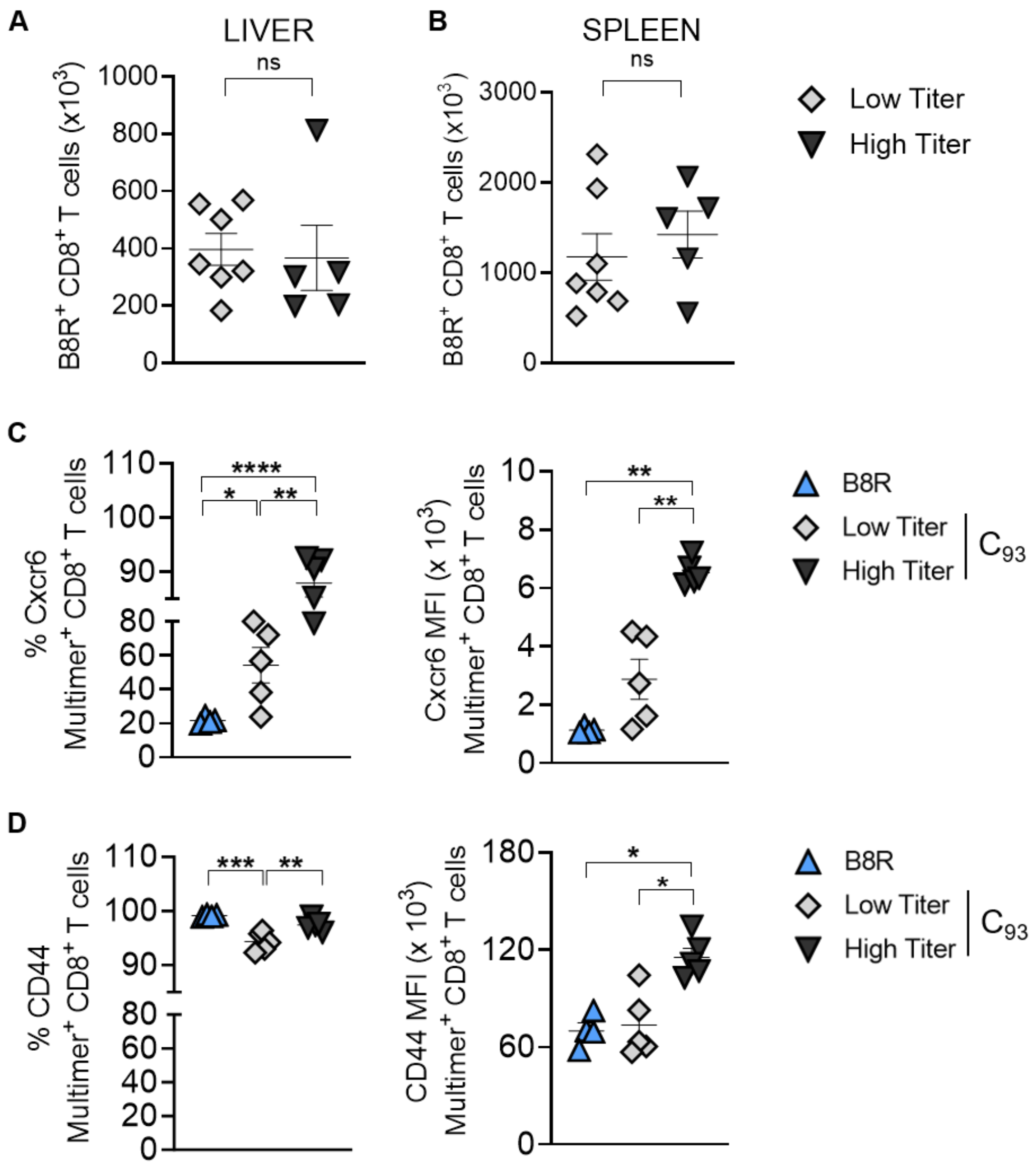

Fig. S2. MVA-specific CD8+ T cells in the liver and spleen at week 5

C57BL/6J mice were intravenously injected with AAV-HBV1.2 six weeks before vaccination to establish low- and high-level persistent HBV replication. Mice were vaccinated intramuscularly following the *TherVacB* regimen. The analyses were performed 1 week after completion of the vaccination scheme, at week 5. Counts of MVA(B8R)-specific (A) liver-associated and (B) splenic $CD8^+$ T cells in low- and high-titer mice. Expression of (C) Cxcr6 and (D) CD44 on B8R- and C_{93}-specific liver-associated $CD8^+$ T cells and their mean fluorescent index (MFI) at week 5. Cell counts were determined using CountBright™ absolute counting beads for flow cytometry after ex vivo multimer staining. Statistical analyses were performed using (A-B) the Mann-Whitney U-test and (C-D) the two-way ANOVA. Asterisks mark statistically significant differences: **p*≤0.05; **p≤0.01, ***p≤0.001; ****p≤0.0001; n.s, not significant. The mean and SEM are shown (n ≥ 4).

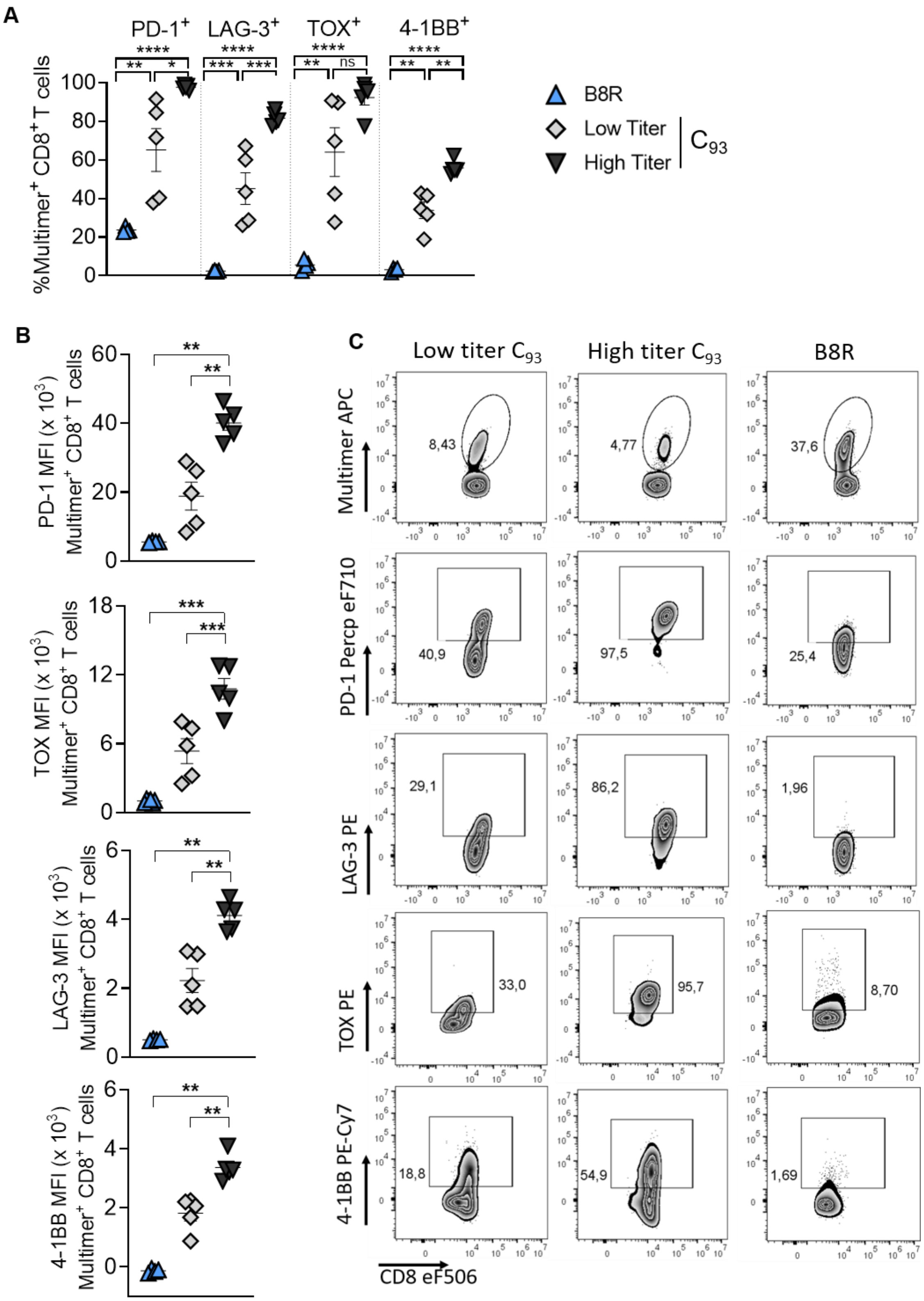

Fig. S3. Comparison of MVA-specific and HBV-specific immune cell marker expression on CD8⁺ T cells at week 5

C57BL/6J mice were intravenously injected with AAV-HBV1.2 six weeks before vaccination to establish low- and high-level persistent HBV replication. Mice were vaccinated intramuscularly

following the *TherVacB* regimen. The analyses were performed 1 week after completion of the vaccination scheme, at week 5. (A-B) Expression of PD-1, LAG-3, TOX, and 4-1BB on MVA(B8R)-specific and C_{93}-specific liver-associated $CD8^+$ T cells and their mean fluorescent index (MFI) at week 5. Cell counts were determined using CountBright™ absolute counting beads for flow cytometry after ex vivo multimer staining. (C) Representative flow cytometry plots of C_{93}-specific and B8R-specific and $CD8^+$ T cells detected with C_{93}- or B8R-specific MHC-I in isolated liver-associated lymphocytes. Statistical analyses were performed using the two-way ANOVA. Asterisks mark statistically significant differences: **p*≤0.05; **p≤0.01, ***p≤0.001; ****p≤0.0001; n.s: not significant. The mean and SEM are shown (n ≥ 4).

Supplementary Materials and Methods

Table S1: Antibodies

Antibody	Conjugated Color	Dilution	Reference Number	Major Supplier
anti-hGzmB	PE	1:100	GRB04	Invitrogen
anti-m4-1BB	PE-Cy7	1:200	25-1371-82	eBioScience
anti-mCD19	AF700	1:100	115528	Biolegend
anti-mCD25	APC	1:100	17-0251-81	eBioscince
anti-mCD3	FITC	1:100	100203	Biolegend
anti-mCD4	AF700	1:100	100536	Biolegend
anti-mCD4	PE	1:200	12-0041-83	eBioscience
anti-mCD4	APC	1:100	17-0041-84	eBioscience
anti-mCD44	PE	1:250	12-0441-82	eBioscience
anti-mCD45.2	PE	1:200	12-0454-83	eBioscience
anti-mCD8a	APC	1:100	17-0041-83	Invitrogen
anti-mCD8a	eF506	1:100	69-0081-82	Invitrogen
anti-mCD8a	Pacific Blue	1:100	558106	BD Biosciences
anti-mCxcr6	FITC	1:100	151108	Biolegend
anti-mFoxP3	FITC	1:100	11-5773-82	Invitrogen
anti-mIFNγ	FITC	1:300	554411	BD Biosciences
anti-mLag3	PE	1:200	12-2231-83	Invitrogen
anti-mLag3	eFluor450	1:100	48-2231-80	eBioscience

anti-mNK1.1	PE-Cy7	1:100	108714	Biolegend
anti-mPD1	PerCp eFluor 710	1:200	46-9985-82	eBioscience
anti-mTNF	PE-Cy7	1:200	557644	BD Biosciences
anti-mTox	PE	1:200	12-6502-82	Invitrogen
Fixable Viability Dye	eFluor780	1:3000	65-0865-18	eBioscience
Strep-Tavidin	APC	1:100	17-4317-82	Invitrogen

Table S2: HBsAg-derived peptide pool

Numbering	Position	Amino acid Sequence
Sp3-37	145-159	GNCTCIPIPSSWAFA
Sp3-38	149-163	CIPIPSSWAFAKYLW
Sp3-39	153-167	PSSWAFAKYLWEWAS
Sp3-40	157-171	AFAKYLWEWASARFS
Sp3-41	161-175	YLWEWASARFSWLSL
Sp3-42	165-179	WASARFSWLSLLVPF
Sp3-43	169-183	RFSWLSLLVPFVQWF
Sp3-44	173-187	LSLLVPFVQWFVGLS
Sp3-45	177-191	VPFVQWFVGLSPTVW
Sp3-46	181-195	QWFVGLSPTVWLSAI
Sp3-47	185-199	GLSPTVWLSAIWMMW
Sp3-48	189-203	TVWLSAIWMMWYWGP
Sp3-49	193-207	SAIWMMWYWGPSLYS
Sp3-50	197-211	MMWYWGPSLYSIVSP
Sp3-51	201-215	WGPSLYSIVSPFIPL
Sp3-52	205-219	LYSIVSPFIPLLPIF
Sp3-53	209-223	VSPFIPLLPIFFCLW
Sp3-54	213-226	IPLLPIFFCLWVYI

Table S3: HBcAg-derived peptide pool

Numbering	Position	Amino acid Sequence
Cp2-188	70-87	TWVGGNLEDPISRDLVVS
Cp2-189	77-94	EDPISRDLVVSYVNTNMG
Cp2-190	84-101	LVVSYVNTNMGLKFRQLL
Cp2-191	91-108	TNMGLKFRQLLWFHISCL
Cp2-192	98-115	RQLLWFHISCLTFGRETV
Cp2-193	105-122	ISCLTFGRETVIEYLVSF
Cp2-194	112-129	RETVIEYLVSFGVWIRTP
Cp2-195	119-136	LVSFGVWIRTPPAYRPPN

Cp2-196	126-143	IRTPPAYRPPNAPILSTL
Cp2-197	133-150	RPPNAPILSTLPETTVVR
Cp2-198	140-157	LSTLPETTVVRRRGRSPR

Table S4: Set of primers and cycling conditions for the HBV-DNA PCR

Primer Name	Sequence
rcDNA Fwd (HBV1745)	5’-GGAGGGATACATAGAGGTTCCTTGA-3’
rcDNA Rev (HBV 1844)	5´-GTTGCCCGTTTGTCCTCTAATTC-3´
mPrp Fwd	5’- GCGGTACATGTTTTCACGGTAGTA-3’
mPrp Rev	5’-GAGCAGGCCCATGATCCA-3’

Cycling conditions: 5 min 95 °C followed by 40× (15 s 95 °C, 10 s 60 °C, 25 s 72°C).

Table S5: The set of primers and cycling conditions for HBV-RNA PCR

Primer Name	Sequence
pgRNA Fwd[1]	5’-GAGTGTGGATTCGCACTCC-3’
pgRNA Rev[1]	5’-GAGGCGAGGGAGTTCTTCT-3’
mGAPDH Fwd[2]	5’-ACCAACTGCTTAGCCC-3’
mGAPDH Rev[2]	5’-CCACGACGGACACATT-3’

Cycling conditions[1]: 5 min 95 °C followed by 40× (15 s 95 °C, 30 s 60 °C).

Cycling conditions[2]: 5 min 95 °C followed by 40× (15 s 95 °C, 10 s 60 °C, 25 s 72 °C).
