# Supplementary material for: High HBV antigen levels drive loss of vaccine-induced CD8+ T-cell function in carrier mice over time
Source: JHEP Rep. 2026 Sep 10;8(12):102033. doi: 10.1016/j.jhepr.2026.102033 (PMC13743956; doi:10.1016/j.jhepr.2026.102033)
Supplement: Supplementary file 3 [file mmc4.pdf]

Research article

High HBV antigen levels drive loss of vaccine-induced CD8+ T-cell function in carrier mice over time

Authors

Edanur Ates Öz, Jinpeng Su, Merve Gültan, …, Percy Knolle, Ulrike Protzer, Anna Kosinska

Correspondence

anna.kosinska@tum.de, anna.kosinska@helmholtz-munich.de (A. Kosinska).

Graphical abstract

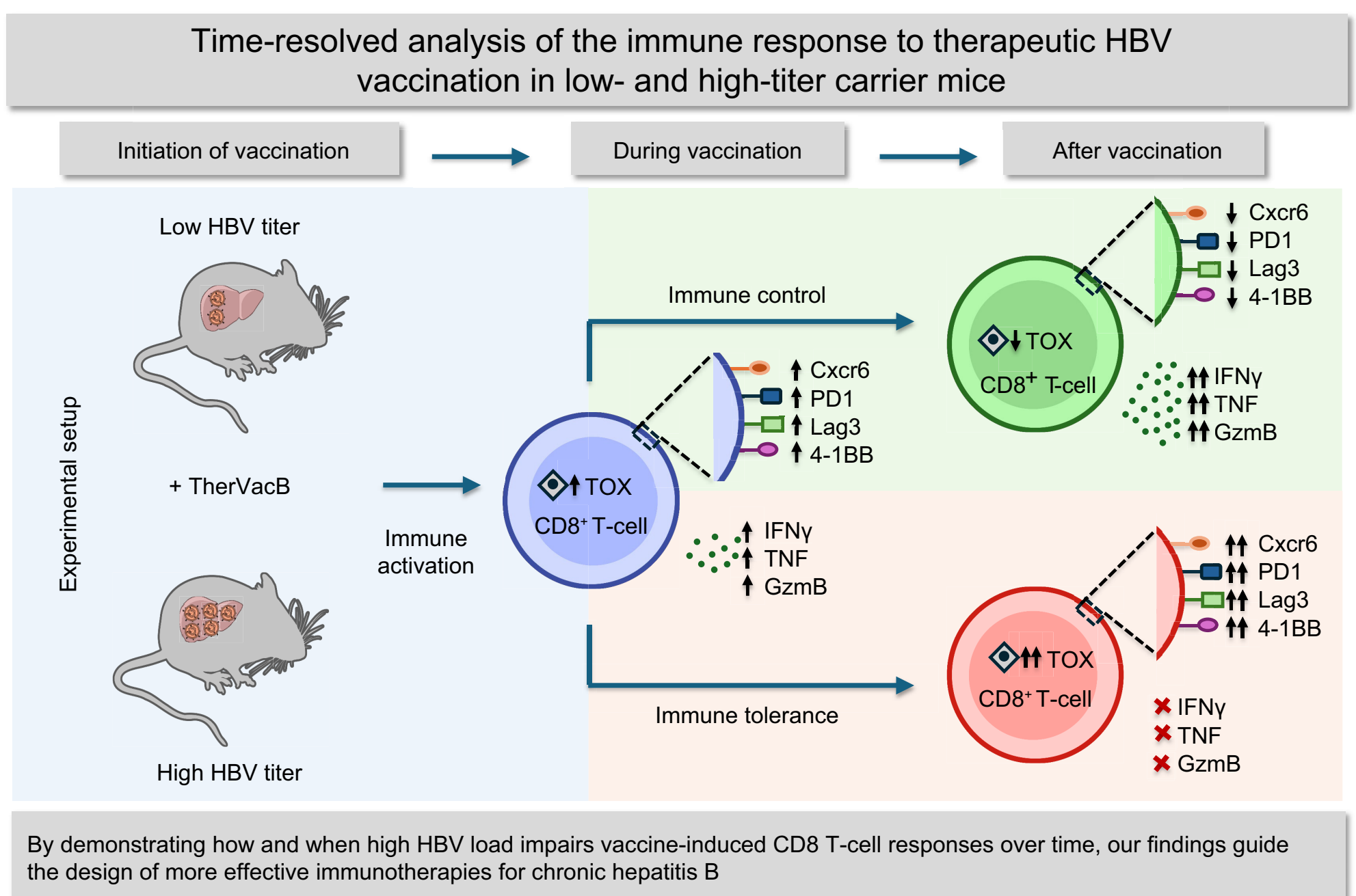

Highlights:

- Baseline HBV antigen load determines durability of vaccine-induced CD8+ T cells.
- Therapeutic vaccination induces CD8+ T-cell infiltration into the liver regardless of titer.
- High antigen load drives CD8+ T-cell dysfunction, deletion, and treatment failure.
- Early immune modulation during the therapeutic vaccination may enhance durable HBV control.

Impact and implications:

This study demonstrates that baseline HBV antigen load critically determines the durability of vaccine-induced CD8+ T-cell immunity. Although therapeutic vaccination initially elicited liver-infiltrating, HBV-specific CD8+ T cells irrespective of the viral titer, only low-titer infection permitted sustained viral clearance and memory T-cell formation. In high-titer HBV carriers, persistent antigen exposure drove progressive T-cell exhaustion and deletion, resulting in transient viral control and treatment failure. Importantly, the data identify a critical time window in which early intervention to prevent or reverse T-cell dysfunction may be decisive. Clinically, these findings support combining therapeutic vaccination with antigen-lowering or immunomodulatory strategies applied early to achieve durable immune control in chronic HBV infection.

https://doi.org/10.1016/j.jhepr.2026.102033
© 2026 The Authors. Published by Elsevier B.V. on behalf of European Association for the Study of the Liver (EASL). This is an open access article under the CC BY license (http://creativecommons.org/licenses/by/4.0/).

Research article

JHEP Reports

High HBV antigen levels drive loss of vaccine-induced CD8+ T-cell function in carrier mice over time

Edanur Ates Öz[1,2], Jinpeng Su[1,2], Merve Gültan[1], Margaret Tulessin[3], Nikolas Müller[1], Hélène Anne Kerth[1], Swati Singh[1], Carolin Mogler[3], Katja Steiger[3], Jochen Wettengel[1,2], Dirk Busch[2,4], Percy Knolle[2,5], Ulrike Protzer[1,2], Anna Kosinska[1,2,*]

JHEP Reports **2026**. vol. 8 | 1–14

Check for updates

Background & Aims: Chronic HBV infection persists because of the dysfunction of virus-specific CD8+ T cells. High HBV antigen levels impair the success of therapeutic vaccination. The influence of viral titers on vaccine-induced immunity remains to be fully elucidated. We compared the kinetics of vaccine responses in low- and high-titer HBV carrier mice to identify tipping points and dissect the decisive steps determining the outcome of therapeutic vaccination.

Methods: We established low- and high-titer persistent HBV replication in the livers of immunocompetent C57BL/6J mice using adeno-associated-virus (AAV)-HBV and administered *TherVacB*, a heterologous protein prime/modified vaccinia Ankara boost vaccine. Serum and liver samples were collected during vaccination and subsequently longitudinally to analyze HBV-specific immune responses and viral parameters.

Results: HBV antigen levels before vaccination did not significantly influence the hepatitis B surface antibody (anti-HBs) response (p >0.9); however, they had a significant effect on the vaccine-induced CD8 T-cell response. *TherVacB* induced activated core-specific CD8+ T cells that efficiently infiltrated the liver in low- and high-titer HBV carriers. Low-titer HBV carrier mice achieved sustained viral clearance and memory T-cell formation, whereas high-titer mice exhibited only transient viral reduction, followed by CD8+ T-cell dysfunction and failure to clear the infection. The persistence of HBV infection and constant exposure to high antigen levels led to the overexpression of exhaustion markers, such as PD-1 and TOX (p <0.01), as well as the co-stimulatory receptor 4-1BB (p <0.01). Core-specific CD8+ T cells remained trapped in the liver, progressively lost their effector function, and were eventually eliminated.

Conclusions: These findings identify critical time points that influence vaccine-induced CD8 T-cell responses, suggesting that early intervention targeting T-cell dysfunction may enhance therapeutic vaccination outcomes in chronic HBV infection.

© 2026 The Authors. Published by Elsevier B.V. on behalf of European Association for the Study of the Liver (EASL). This is an open access article under the CC BY license (http://creativecommons.org/licenses/by/4.0/).

Introduction

Chronic HBV infection affects 254 million people worldwide. It is associated with a high risk of developing HBV-associated liver cirrhosis and hepatocellular carcinoma, leading to 1.1 million deaths annually.[1] Currently available treatments for chronic hepatitis B with pegylated-interferon-α or nucleos(t)ide analogs are either inapplicable for many patients or rarely achieve HBV cure.[2,3]

Chronic HBV infection is associated with inadequate activation of both innate and adaptive immunity, resulting in a scarcity and dysfunction of virus-specific CD8+ T cells that are unable to eliminate infected hepatocytes and clear the infection.[4,5] In contrast, acute HBV infection—and, in sporadic cases, chronic HBV infection—is resolved by effective, multi-specific virus-specific CD8 T-cell responses.[6] Therefore, activating the patient's immune system with a potent therapeutic vaccine to control chronic HBV infection is a promising strategy for achieving a cure for HBV.[5]

We developed a therapeutic hepatitis B vaccine, *TherVacB*, which follows a heterologous protein prime/recombinant modified vaccinia Ankara (MVA) boost scheme[7] that is currently under evaluation in clinical trials. For prime immunization, recombinant HBV S and core antigens, which form virus-like particles (VLPs), are used to induce neutralizing antibodies that capture circulating HBsAg, prevent virus spread, and prime HBV-specific CD4+ and CD8+ T cells. The subsequent boost with recombinant MVA, designed to target 95% of circulating HBV isolates, aims to expand virus-specific CD8 T-cell responses.[8–10] In preclinical mouse models with low to intermediate HBV levels, *TherVacB* elicited strong HBV-specific immune responses and long-term immune control of HBV.[8,11] However, high levels of HBV antigens impaired vaccine-induced HBV-specific CD8 T-cell response and anti-

* Corresponding author. Address: Institute of Virology, Technical University of Munich/Helmholtz Munich, Trogerstrasse 30, 81675, Munich, Germany. Tel.: +49 8941406886; fax: +49 8941407359.

E-mail addresses: anna.kosinska@tum.de, anna.kosinska@helmholtz-munich.de (A. Kosinska).

https://doi.org/10.1016/j.jhepr.2026.102033

viral efficacy. Reducing HBV antigen levels using siRNA targeting HBV improved the vaccine efficacy, indicating that low antigen levels are required for effective CD8 T-cell response and viral clearance.[12,13] Subsequent studies have demonstrated that the level of antigen expressed in the liver, rather than circulating antigen levels, is decisive for T-cell function.[14,15] Nevertheless, in clinical studies, siRNA-mediated HBV reduction alone had only limited effects on restoring HBV-specific immunity,[16] highlighting that additional mechanisms contribute to vaccine unresponsiveness in high-titer HBV carriers. This remains to be fully elucidated.

In this study, we longitudinally compared antiviral immune responses induced by therapeutic vaccination in low- and high-titer HBV carrier mice across the consecutive stages of immunization, with a particular focus on liver-associated virus-specific CD8 T-cell responses. We confirmed that persistent exposure to high antigen levels is a decisive determinant of vaccine efficacy. Our immunologic and viral analyses revealed the kinetics of HBV-specific CD8$^+$ T-cell expansion, functional loss, and upregulation of inhibitory markers in high-titer infection. By time-resolved analysis of CD8$^+$ T-cell impairment, we identified a critical window for therapeutic intervention that could enhance the efficacy of therapeutic vaccination in high-titer carriers.

Material and methods

Ethical statements and animal experiments

Animal experiments were conducted in accordance with the German regulations of the Society for Laboratory Animal Science (GV-SOLAS) and the European Health Law of the Federation of Laboratory Animal Science Associations (FELASA) and were reported according to the ARRIVE (Animal Research: Reporting of *in vivo* Experiments) guidelines. Experiments were approved by the District Government of Upper Bavaria (permission number: ROB-55.2-2532.Vet_02-18-24). Mice were housed under biosafety level 2 and specific pathogen-free conditions, in accordance with institutional guidelines.

Eight-to ten-week-old male C57BL/6J mice were purchased from Janvier Labs (Le Genest-Saint-Isle, France) and acclimatized for at least 7 days. To establish persistent low- and high-titer persistent HBV replication, mice were intravenously injected with 2×10^9 or 1×10^{10} genome equivalents of the adeno-associated-virus (AAV)-HBV1.2 vector encoding a 1.2-fold over-length HBV genome of genotype D (AAV-HBV) 6 weeks before vaccination. Low-titer mice were defined by an HBsAg level of 133.9 (±69.7) IU/ml, whereas high-titer mice were defined by an HBsAg level of 1093.8 (±665.2) IU/ml at baseline. The animals' overall well-being and weight were monitored weekly. The group size (n ≥4) was determined by a statistician to ensure adequate power for reliable statistical analyses.

Heterologous protein prime/MVA boost vaccination regimen

Mice received the therapeutic heterologous protein prime/MVA boost vaccine (*TherVacB*) as previously described.[7] In brief, each mouse was intramuscularly injected into both hind limbs with a dosage of 10 μg each of VLPs consisting of HBV S (genotype A; Biovac, Cape Town, South Africa) and core (genotype D; APP Lativijas Biomedicinas, Riga, Latvia) proteins formulated with 10 μg of c-di-AMP as an adjuvant (InvivoGen, San Diego, CA, USA) at Weeks 0 and 2. A booster immunization was administered at Week 4, with each mouse receiving an intramuscular injection of 3×10^7 infectious units of recombinant MVA expressing the corresponding HBV S and core proteins (MVA-S and MVA-core, respectively).[17]

Isolation of splenocytes and liver-associated lymphocytes and flow cytometry analysis

Single-cell suspensions of murine splenocytes and liver-associated lymphocytes (LALs) were isolated as previously described.[7,12]

HBV-specific CD8$^+$ T cells were detected through staining with MHC class I multimers conjugated with the H-2^{Kb}-restricted HBV-specific $S_{190–197}$ (S_{190}; VWLSAIWM) or $core_{93–100}$ (C_{93}; MGLKFRQL). MVA-derived multimer $B8R_{20–27}$ (MVA_{B8R}; TSYKFESV) was used as a control. Multimers were prepared as previously described.[18] Before use, multimers were labeled with streptavidin-APC (Thermo Fisher Scientific, Waltham, MA, USA) as previously described.[12] Cell surface staining was performed using anti-CD4, anti-CD8, anti-LAG3, anti-PD1, anti-4-1BB, anti-CXCR6, and anti-CD44 antibodies (Table S1). The lymphocyte populations were characterized using anti-CD45.2, anti-CD3, anti-CD19, anti-CD4, anti-CD8, anti-NK1.1, and anti-CD25 antibodies (Table S1). Dead cells were excluded from analysis by staining with Fixable Viability Dye eF780 (Thermo Fisher Scientific). For intranuclear staining, cells were washed and fixed using the FOXP3 Fixation/Permeabilization kit (Thermo Fisher Scientific) according to the manufacturer's instructions. Cells were then permeabilized for 4 h and stained intranuclearly with anti-TOX or anti-FOXP3 antibodies.

For intracellular cytokine staining (ICS), splenocytes and LALs were stimulated overnight with HBsAg-derived (Table S2) and HBcAg-derived (Table S3) overlapping peptide pools[9] or with the control OVA_{S8L} peptide (SIINFEKL). Cell surface staining was performed using anti-CD4 and anti-CD8 antibodies (Table S1). interferon γ (IFNγ), tumor necrosis factor (TNF), and granzyme B (GzmB) were detected intracellularly as previously described.[7]

Absolute cell numbers were determined using CountBright^{TM} counting beads (Thermo Fisher Scientific) according to the manufacturer's instructions. Data were acquired using a CytoFlexS flow cytometer (Beckman Coulter, Indianapolis, IN, USA) and analyzed using FlowJo software v10.8 (Tree Star, Woodburn, OR, USA).

Serological and viral analyses

Serum HBsAg, HBeAg, hepatitis B e antibody (anti-HBe), and hepatitis B surface antibody (anti-HBs) levels were quantified on an Architect^{TM} platform (Abbott, Wiesbaden, Germany), and hepatitis B core antibody (anti-HBc) levels were measured using the LIAISON® (DiaSorin, Saluggia, Italy) as previously described.[7] Serum alanine aminotransferase (ALT) activity was determined using the Reflotron® GPT/ALT tests (Roche Diagnostics, Mannheim, Germany) or the Respons910 ALAT/GPT kit (DiaSys, Holzheim, Germany) according to the manufacturer's instructions.

H&E staining of deparaffinized liver sections was performed according to standard protocols. Histopathological evaluation and inflammation scoring were performed as previously described, using the Desmet grading system,[19] which has been adapted for murine hepatitis. For immunohistochemistry, formalin-fixed liver sections were stained with an anti-HBcAg antibody (LS Bio, Newark, CA, USA; 1:50 dilution) as previously described.[13] The number of core-positive hepatocytes was determined in 10 randomly selected fields at 20 × magnification and quantified per square millimeter (mm^2).

Intrahepatic HBV DNA and RNA were extracted from 20 and 30 mg of liver tissue, respectively, using Nucleospin Tissue kits (Macherey-Nagel, Düren, Germany) according to the manufacturer's instructions. HBV DNA (rcDNA) was quantified using PCR with SYBR Green on a LightCycler instrument (Roche Diagnostics, Rotkreuz, Switzerland) and normalized to the Prp gene (Table S4). The 3.5-kb pregenomic RNA (pgRNA) was quantified using real-time PCR with SYBR Green and normalized to GAPDH (Table S5).

HBV neutralization assay

HBV neutralization assay was performed as previously described.[20–22] In brief, HepG2-NTCP (in-house produced) cells were seeded at 70–80% confluency in 2.5% DMSO (Merck, Darmstadt, Germany) 3 days before infection with purified NanoLuc-expressing HBV (rHBV). Pooled mouse sera from each vaccination group were serially diluted in medium with 2.5% DMSO and 4% PEG-6000 (Merck), mixed with rHBV, and incubated for 30 min at room temperature. Cells were inoculated overnight, washed with PBS, and cultured in fresh medium with 2.5% DMSO. Supernatants were collected on day 7 after infection. Luminescence was measured using an Infinite F200 Tecan plate reader (Tecan Group, Männedorf, Switzerland) with 10 µl of supernatant and 100 µl of substrate solution (1 µM coelenterazine dissolved in acidified methanol, diluted 1:1,000 in PBS supplemented with 0.1% Tween-20; PJK, Kleinblittersdorf, Germany). Inhibition of infection was normalized to naïve serum controls, and half-maximal inhibitory concentration (IC_{50}) values were determined using four-parameter nonlinear regression in GraphPad Prism (GraphPad Software Inc., San Diego, CA, USA).

Statistical analyses

In all graphs, data are presented as mean ± SEM. Analyses were conducted using GraphPad Prism version 9.5 or 10.1 (GraphPad Software Inc.), applying one- or two-way ANOVA or the Mann-Whitney *U* test. Values of $p \leq 0.05$ were considered significant, and only statistically significant differences are shown.

Results

High HBV titers prevent HBsAg and HBeAg seroclearance after therapeutic vaccination

To dissect differences in *TherVacB*-mediated antiviral responses between mice with low and high HBV levels, we performed a longitudinal, time-resolved analysis of HBV-related serological parameters during and after therapeutic vaccination. Eight-to ten-week-old C57BL/6J male mice were infected with different AAV-HBV doses to establish low- and high-level persistent HBV replication. Subsequently, the mice were immunized with adjuvanted core- and S-VLPs at Weeks 0 and 2 and boosted with MVA expressing HBV core and S proteins at Week 4 (Fig. 1A). Analyses were performed before initiating *TherVacB* (Week 0), during the immunizations (Weeks 2 and 3), and early (Week 5) and later (Weeks 8, 12, and 18) after completing the vaccination scheme.

With each vaccination, antigen levels decreased in both low- and high-titer mice, reaching their lowest values at Week 5, that is, 1 week after the MVA boost (Fig. 1B). Although in low-titer HBV carriers HBsAg levels remained below the detection limit, HBsAg levels quickly rebounded in high-titer mice, resulting in only a 1-$\log_{10}$ lower level at Week 18 than at baseline. Nevertheless, the amount of anti-HBs antibodies induced was comparable between low- and high-titer mice over the monitoring period (Fig. 1C). To further investigate whether vaccine-induced anti-HBs differ in their neutralization capacity between low- and high-titer mice, we performed a neutralization assay in HepG2-NTCP cells using reporter HBV expressing NanoLuc luciferase. Although sera from low-titer mice demonstrated approximately fivefold stronger HBV neutralization than the sera from high-titer mice at Week 3, this difference was not sustained at Week 5 (Fig. 1D) or Week 18 (Fig. S1). As with anti-HBs antibodies, anti-HBc antibodies were first detected after the second protein vaccination at Week 3 (Fig. 1C,E), indicating that at least two vaccinations are required to induce effective antibody responses. In low-titer mice only, anti-HBc levels further increased after the MVA boost (Fig. 1E).

In contrast to patients with chronic hepatitis B, serum HBeAg is not spontaneously cleared in AAV-HBV-transduced mice; HBeAg seroconversion indicates the elimination of persistent HBV infection.[7,12] Low-titer HBV carriers seroconverted to anti-HBe already after the second protein vaccination (Fig. 1F). Consequently, HBeAg dropped below the detection limit in low-titer mice after the MVA boost (Fig. 1G), which was preceded by a transient serum alanine transaminase (sALT) flare, indicating the elimination of HBV-positive hepatocytes (Fig. 1H). In contrast, high-titer mice showed no detectable increase in ALT except at Week 2, suggesting that the short-term antiviral effect may be induced even in the presence of high HBV levels.

High HBV titers prevent efficient elimination of the infected hepatocytes after therapeutic vaccination

To further substantiate our findings and confirm the effects of *TherVacB* on HBV infection status in low-*vs*. high-titer mice, we assessed liver inflammation and analyzed viral parameters in liver tissue collected at consecutive time points.

Although we observed immune cell infiltration into the liver parenchyma in both low- and high-titer mice following *the TherVacB* vaccination (Fig. 2A), no significant histopathological changes were detected, with only minimal or mild inflammation scores (Fig. 2B). Although low-titer mice exhibited a gradual decrease in the number of core-positive hepatocytes during vaccination, ultimately becoming negative after completion of the entire *TherVacB* scheme, the number of HBV-positive hepatocytes remained high throughout the monitoring period in high-titer mice (Fig. 2C,D). Although the clearance of HBV from the liver took longer than from the blood, *TherVacB* ultimately

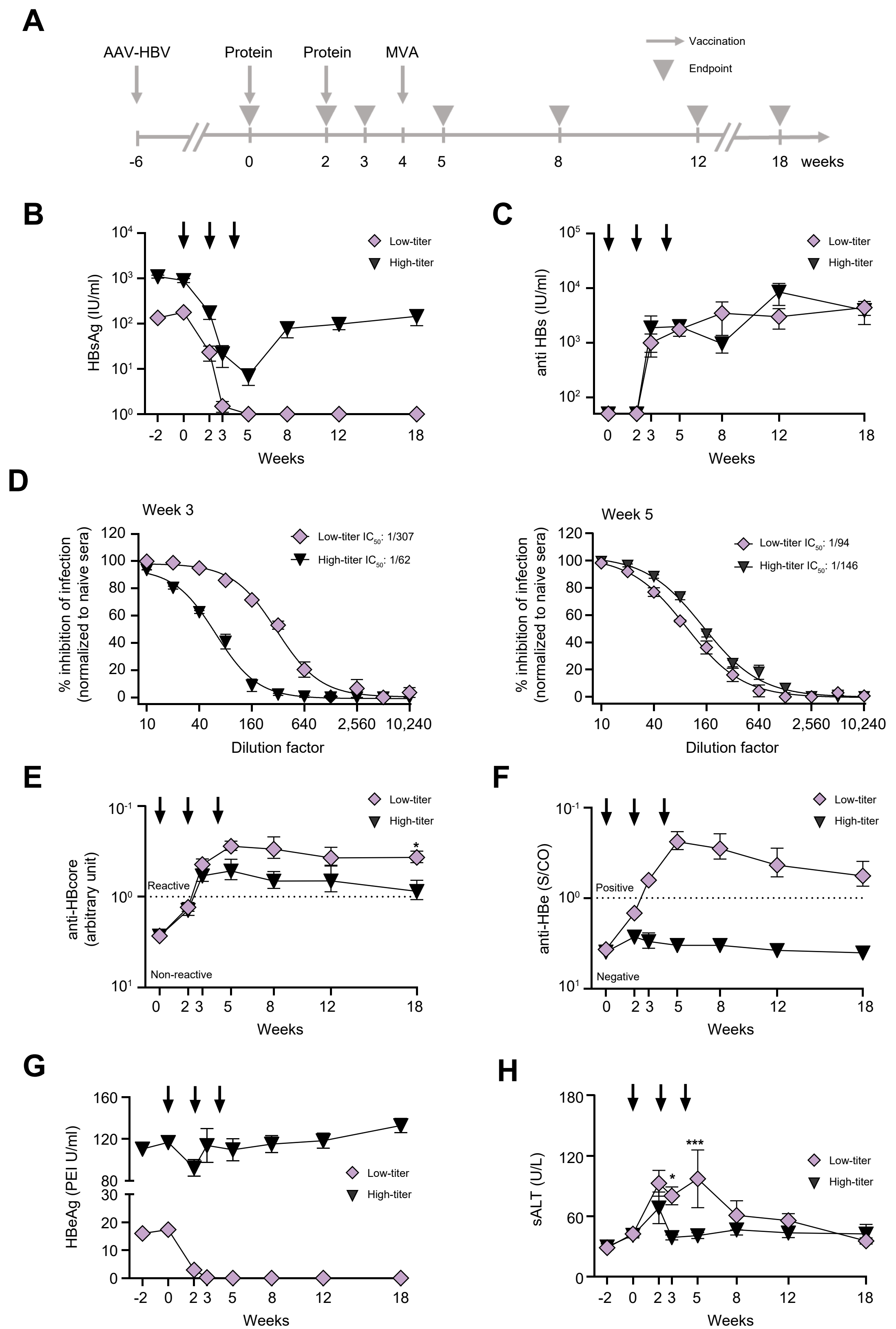

Fig. 1. Effect of *TherVacB* on the HBV-related serological parameters. (A) C57BL/6J mice were intravenously injected with AAV-HBV1.2 6 weeks before vaccination to establish low- and high-level persistent HBV replication. Mice were then immunized intramuscularly (black arrows) following the *TherVacB* regimen, twice using a combination of adjuvanted HBV S- and core-VLPs (Weeks 0 and 2) (10 µg each) and once with MVA expressing HBV-core and –S proteins (Week 4) (3 × 10^7 IFU each). The final analyses were performed at the indicated time points (gray triangles). Time kinetics of serum (B) HBsAg and (C) anti-HBs in low-*vs*. high-titer

resulted in the clearance of HBV DNA and pgRNA in low-titer mice by Week 18 (Fig. 2E,F). Interestingly, the amount of intrahepatic HBV DNA and pgRNA decreased at Week 2 in high-titer mice but eventually rebounded to almost the starting level by Week 18 (Fig. 2E,F).

These results demonstrate that *TherVacB* rapidly eliminates HBV from the livers of low-titer carriers but fails to do so in high-titer HBV carrier mice.

Therapeutic vaccination triggers lymphocyte infiltration into the liver

To investigate how *TherVacB* modulates the dynamics of the immune response in low-*vs*. high-titer HBV carriers in the secondary lymphoid organs, the spleen, and the site of infection, the liver, we compared counts of relevant immune cell populations during and after vaccination.

Six weeks after establishing low- and high-titer persistent HBV replication, before we applied *TherVacB* (Week 0), there were no significant differences in the counts of B cells, $CD4^+$ and $CD8^+$ T cells, and natural killer (NK) cells between the groups of mice, in either the spleen or the liver (Fig. 3A–D). One week after the complete *TherVacB*, at Week 5, the numbers of all examined effector cell populations increased substantially, regardless of the initial infection levels. The only differences observed were higher $CD8^+$ T cell counts (Fig. 3C) and NK cell counts (Fig. 3D) in the spleen at Week 5 and B cell counts in the liver at Week 18 (Fig. 3A) in high-titer mice, whereas the counts of NK cells were significantly increased in the livers of low-titer mice at Week 5 (Fig. 3D).

Given that high-titer mice were unable to resolve persistent HBV infection after *TherVacB*, we next investigated whether regulatory T cells (Tregs) could play a role in suppressing vaccine-mediated immunity in high-titer HBV carriers. We compared the numbers of Tregs between low- and high-titer carriers but found no difference in the counts of splenic or intrahepatic Tregs at any of the time points examined (Fig. 3E).

These data demonstrate that *TherVacB* resulted in rapid expansion of the major immune cell populations in the spleens and livers of all immunized mice, with only subtle differences in the counts of hepatic NK and B cell populations that could be detected between low- and high-titer mice. Moreover, our data suggest that Tregs are unlikely to limit the efficacy of *TherVacB* in high-titer AAV-HBV mice.

Vaccine-induced HBV-specific $CD8^+$ T cells experience different fates depending on the initial HBV levels

Given that HBV-specific $CD8^+$ T cells are key to viral clearance,[23] we examined how HBV infection levels shape vaccine-induced $CD8^+$ T-cell immunity during and after vaccination. We first compared the numbers of HBV core- and S-specific $CD8^+$ T cells in the livers and spleens of low- and high-titer HBV carriers, using C_{93} and S_{190} MHC class I multimers, respectively.

In the liver, counts of C_{93}-specific $CD8^+$ T cells steadily increased after each protein immunization in low- and high-titer HBV carriers, achieving similar numbers at Weeks 2 and 3 (Fig. 4A), indicating that *TherVacB* is capable of expanding HBV-specific $CD8^+$ T cells in both settings. In low-titer HBV carriers, intrahepatic C_{93}-specific $CD8^+$ T cells vigorously expanded following MVA booster vaccination, reaching peak levels at Week 5. Following HBV elimination, the C_{93}-specific $CD8^+$ T cell population contracted but then stabilized (Fig. 4A). In high-titer carriers, by contrast, C_{93}-specific $CD8^+$ T cells did not further expand after MVA boost vaccination, but instead gradually declined in numbers and were barely detectable by Week 18. In low-titer carriers, C_{93}-specific $CD8^+$ T cells became detectable in the spleen at Week 5, correlating with the resolution of persistent HBV infection (Fig. 4B). In the spleens of high-titer carriers, no C_{93}-specific $CD8^+$ T cells could be detected at any time point. Counts of MVA-specific $CD8^+$ T cells were comparable between low- and high-titer carriers and readily detected in spleen and liver (Fig. S2A and B), demonstrating that the observed differences in numbers of C_{93}-specific $CD8^+$ T cells were strictly HBV specific.

Surprisingly, S_{190}-specific CD8 T cells were detected only after a complete vaccination scheme at Week 5 in both low- and high-titer HBV carrier mice (Fig. 4C,D). The number of S_{190}-specific $CD8^+$ T cells in the livers and spleens of low- and high-titer carriers remained significantly lower than that of C_{93}-specific cells and, interestingly, showed only minor differences between both groups.

We next investigated how different levels of pre-existing HBV replication influence the activation and memory formation of liver-infiltrating vaccine-elicited HBV-specific $CD8^+$ T cells. Because the number of S_{190}-specific $CD8^+$ T cells in both groups of mice was too low to allow a reliable phenotypic analysis, we focused instead on C_{93}-specific $CD8^+$ T cells associated with the liver. We analyzed the expression of Cxcr6, a chemokine receptor that helps guide $CD8^+$ T cells to the liver,[24,25] and found significantly more $Cxcr6^+$ T cells in livers with high HBV antigen expression (Fig. 4E). Interestingly, not only the fraction of Cxcr6-positive C_{93}-specific $CD8^+$ T cells but also the expression levels of Cxcr6 per cell (mean fluorescent index [MFI]) were significantly higher in high-titer than in low-titer HBV carriers (Fig. 4E). In contrast, we found a very low frequency of $Cxcr6^+$ MVA-specific $CD8^+$ T cells (Fig. S2C).

We found that almost all *TherVacB*-induced C_{93}-specific $CD8^+$ T cells in the liver showed an effector/effector memory phenotype ($CD44^+CD62L^-$) in both groups of mice. The expression of activation marker CD44 on C_{93}-specific $CD8^+$ T cells isolated from high-titer carriers increased at Weeks 5 and 8 (Fig. 4F and Fig. S2D). Most of the C_{93}^+ $CD8^+$ T cells co-expressed Cxcr6 and CD44, particularly in high-titer HBV carrier mice, suggesting an enrichment of activated liver-resident $CD8^+$ T cells upon vaccination (Fig. 4G). After clearance of HBV in low-titer carriers, almost all C_{93}-specific $CD8^+$ T

HBV carrier mice. (D) The percent inhibition of infection sera from low- and high-titer mice collected at Weeks 3 and 5 was determined by a neutralization assay using recombinant HBV expressing NanoLuc luciferase. IC_{50} values determined from neutralization curves are shown. Time kinetics of serum (E) anti-HBc, (F) anti-HBe, (G) HBeAg, and (H) sALT levels in low-*vs*. high-titer HBV carrier mice. Statistical analyses were performed using two-way ANOVA. Asterisks mark statistically significant differences: *$p \leq 0.05$; ***$p \leq 0.001$. Data are presented as mean and SEM (n ≥5). AAV, adeno-associated virus; anti-HBs, hepatitis B e antibody; IFU, infectious units; MVA, modified vaccinia Ankara; sALT, serum alanine transaminase; *TherVacB*, therapeutic hepatitis B vaccine; VLP, virus-like particle.

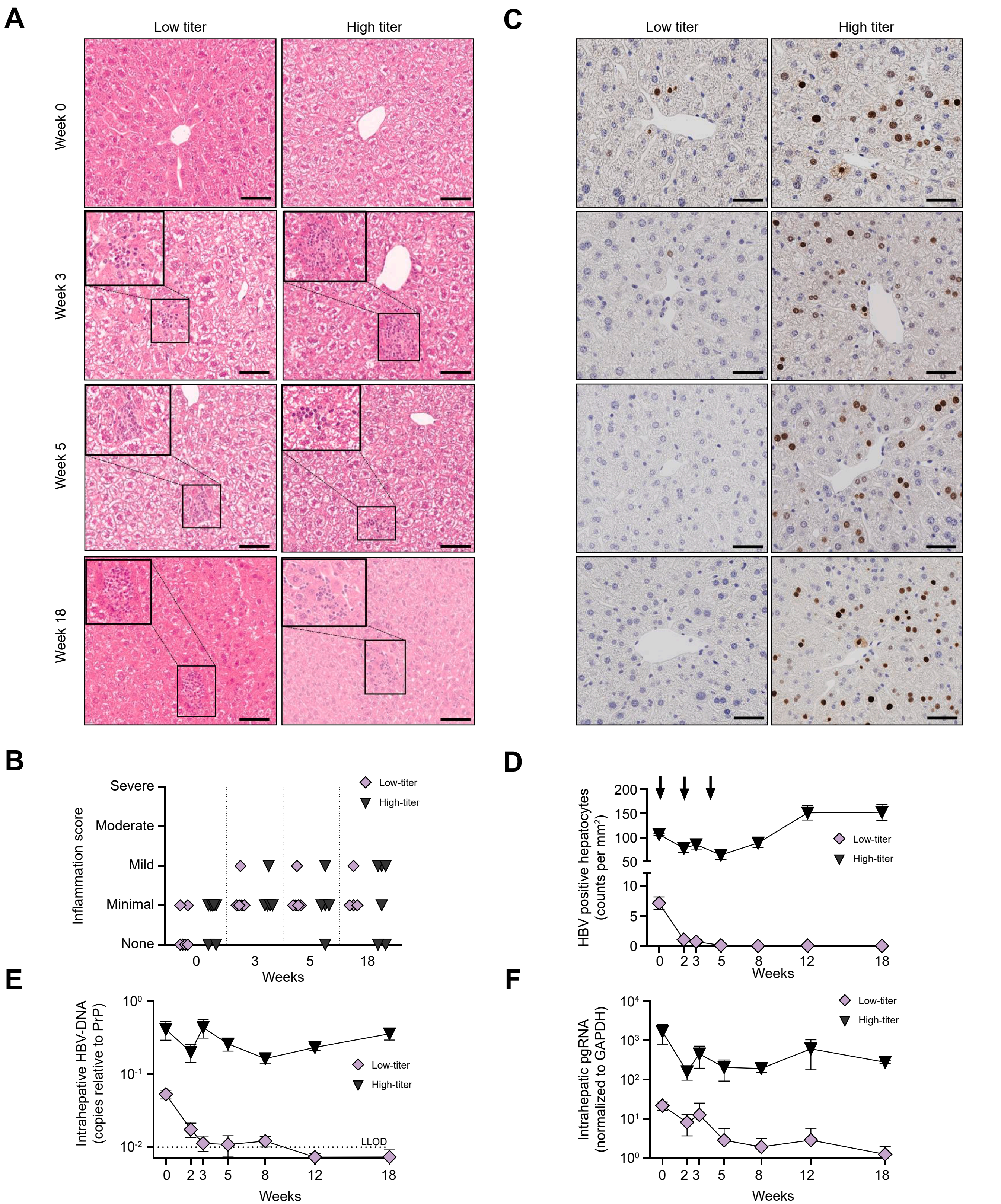

Fig. 2. Effect of *TherVacB* on the HBV persistence in the liver. High- and low-titer HBV carriers were immunized with *TherVacB*, as shown in Fig. 1A; analyses were performed at the indicated time points. Representative images of murine liver (A) H&E staining and (B) histological scoring to assess liver inflammation in individual mice. (C) immunohistochemistry staining and (D) quantification (counts/mm^2) of HBV core-positive hepatocytes (brown nucleus) at the endpoints. Intrahepatic (E) HBV DNA detected in murine liver tissue lysates by qPCR and normalized to the Prp and (F) pgRNA detected in liver tissue lysates by qPCR and normalized to the GAPDH. Data are presented as mean and SEM (n ≥4). pgRNA, pregenomic RNA; qPCR, quantitative PCR; *TherVacB*, therapeutic hepatitis B vaccine.

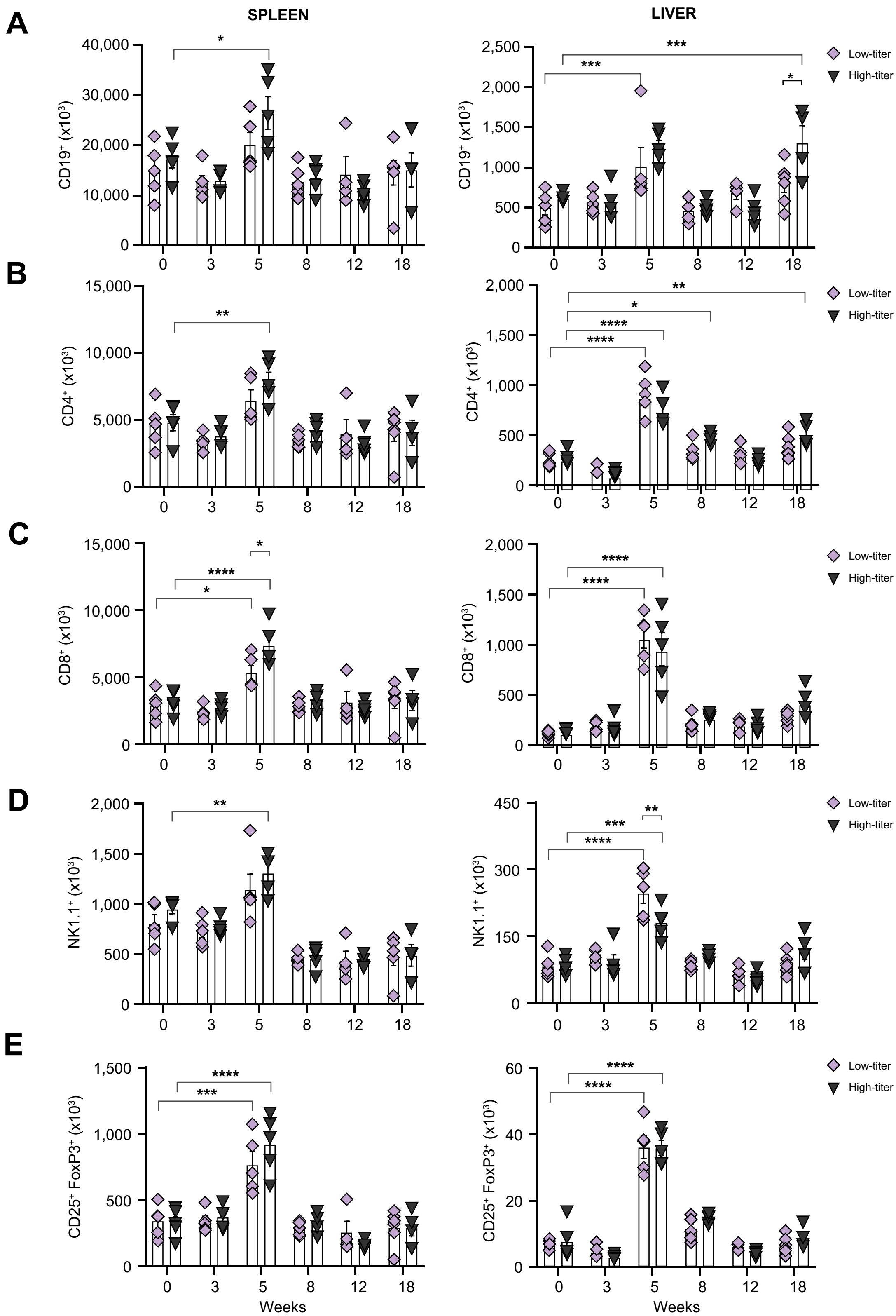

Fig. 3. Changes in lymphocyte numbers in the spleen and liver during and after *TherVacB* vaccination. High- and low-titer HBV carriers were immunized with *TherVacB*, as shown in Fig. 1A; analyses were performed at the indicated time points. Numbers of (A) B, (B) $CD4^+$ T, (C) $CD8^+$ T, (D) NK, and (E) Treg cells in the spleen and the liver detected by multicolor flow cytometry. Cell counts were determined using CountBright™ absolute counting beads. Statistical analyses were conducted using one-way or two-way ANOVA, comparing groups at each independent time point and with Week 0. Asterisks mark statistically significant differences: *p ≤0.05; **p ≤0.01; ***p ≤0.001; ****p ≤0.0001. Data are presented as e mean and SEM (n ≥4). NK, natural killer; *TherVacB*, therapeutic hepatitis B vaccine; Treg, regulatory T cell.

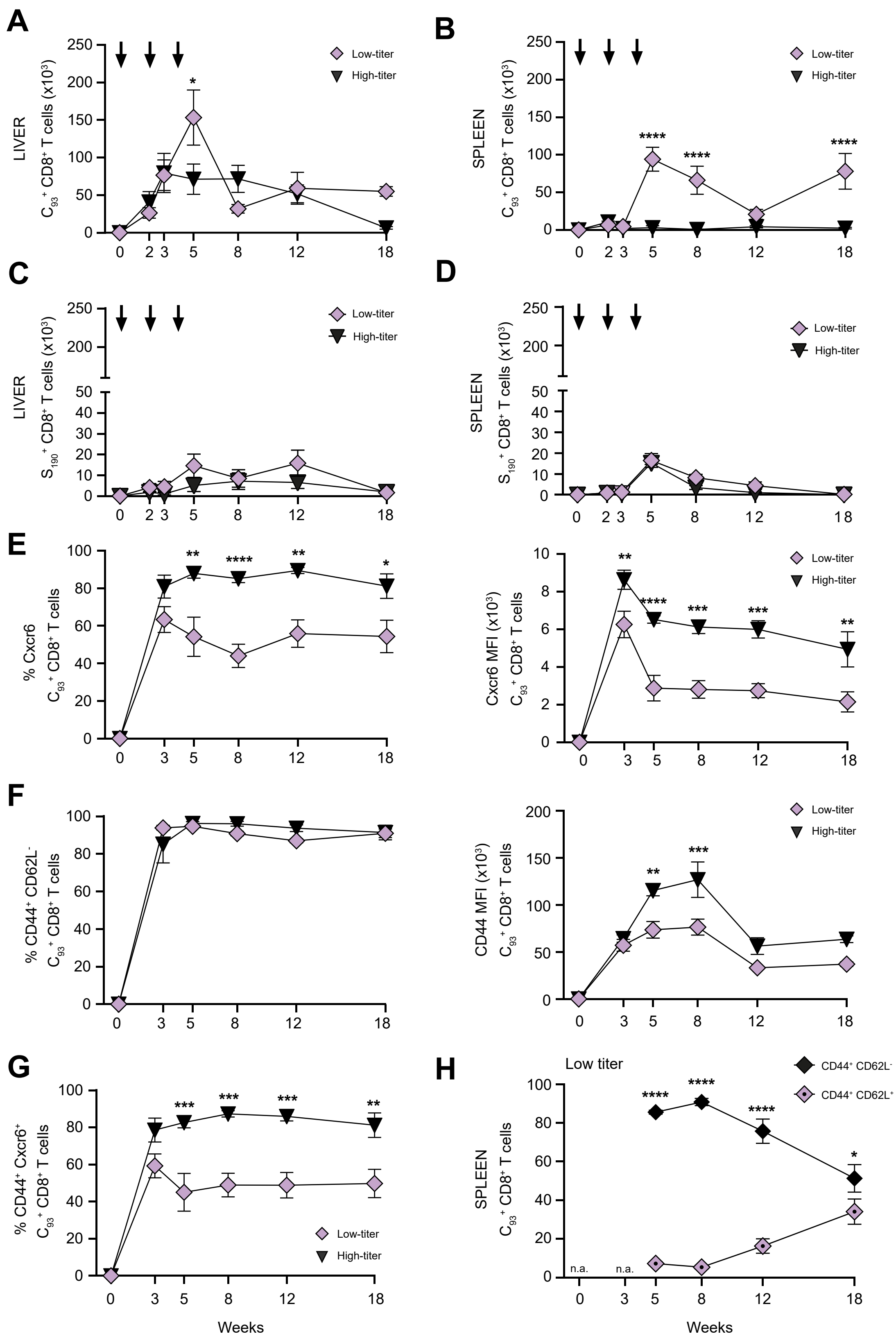

Fig. 4. Phenotypic analysis of *TherVacB*-induced CD8+ T cells. High- and low-titer HBV carriers were immunized with *TherVacB*, as shown in Fig. 1A; analyses were performed at the indicated time points. Numbers of (A) liver-associated and (B) splenic core-specific and (C) liver-associated and (D) splenic S-specific CD8 T cells in low- and high-titer mice detected by staining with C_{93} and S_{190} multimers *ex vivo*. Cell counts were determined using CountBright™ absolute counting beads. Frequencies of C_{93}-specific liver-associated (E) Cxcr6+ and (F) CD44+ CD62L- CD8+ T cells and their MFIs. (G) Frequencies of C_{93}-specific liver-associated

cells detected in the spleen initially showed an effector-memory phenotype, but the proportion of central-memory T cells steadily increased (Fig. 4H).

Taken together, these results demonstrate that *TherVacB* initially induced comparable numbers of HBV-specific effector CD8$^+$ T cells in low- and high-titer HBV carrier mice. The MVA boost only increased the numbers of intrahepatic C_{93}-specific CD8$^+$ T cells in low- but not in high-titer HBV carriers. Liver-associated C_{93}-specific CD8$^+$ T cells showed an effector phenotype and expressed the liver-residency marker Cxcr6. In the presence of high HBV antigens, these cells remained in the liver and were gradually eliminated over time, whereas those in low-titer carrier mice established a memory phenotype.

Vaccine-induced HBV-specific CD8$^+$ T cells from high-titer HBV carriers lose their effector functions over time

Although *TherVacB* efficiently activated substantial numbers of liver-associated HBV-specific CD8$^+$ T cells in high-titer carriers, it still failed to eliminate persistent HBV infection. Thus, we examined how the effector functions of *TherVacB*-induced HBV-specific CD8$^+$ T cells were modulated by low and high HBV levels over time. To dissect the critical time points influencing HBV clearance, we analyzed HBV core- and S-specific IFNγ, TNF, and GzmB production using ICS.

Shortly after the first and second protein immunizations, at Weeks 2 and 3, we found low but comparable numbers of core-specific IFNγ^+, TNF$^+$, and GzmB$^+$ CD8$^+$ T cells in the livers of both low- and high-titer HBV carrier mice (Fig. 5A). At Week 5, following booster vaccination, the numbers of IFNγ^+ CD8$^+$ T cells reached their highest values in the low-titer mice, whereas in the high-titer mice, the numbers of functional core-specific CD8$^+$ T cells decreased and became undetectable at subsequent time points. In low-titer HBV carriers, a strong and multifunctional core-specific CD8 T-cell response was detected in the spleens after Week 5, characterized by high numbers of IFNγ^+, TNF$^+$, and GzmB$^+$ CD8$^+$ T cells (Fig. 5B).

Low-titer mice exhibited a transient effector S-specific CD8 T-cell response, albeit at a significantly lower level than the core-specific response, with IFNγ^+ cells detected in the liver and spleen at Weeks 5 and 8, whereas GzmB$^+$ cells were found only in the liver at Week 5 (Fig. 5C,D). Functional *TherVacB*-induced S-specific CD8$^+$ T cells could not be detected in the livers or spleens of high-titer carrier mice at all.

Taken together, these results indicate that vaccine-elicited CD8$^+$ T cells were initially functional in the livers of high-titer mice but lost their ability to produce antiviral cytokines after the MVA boost. In contrast, functional CD8$^+$ T cells were readily detected in the livers and spleens of low-titer HBV carriers that cleared HBV after vaccination. The detection of only low S-specific CD8 T-cell responses, even in low-titer carriers, suggested a primary role for core-specific CD8$^+$ T cells in resolving the infection.

TherVacB-induced HBV-specific CD8$^+$ T cells exhibit differences in the expression of immune checkpoint markers

We hypothesized that the subsequent loss of effector functions of C_{93}-specific CD8$^+$ T cells in the livers of high-titer carriers may be attributed to either T-cell exhaustion or the upregulation of markers reported to mediate T-cell dysfunction in response to antigen stimulation in the liver. We analyzed liver-associated C_{93}-specific CD8$^+$ T cells for their expression of the exhaustion-related transcription factor TOX, immune checkpoint receptors PD-1 and LAG-3,[26,27] and the co-stimulatory molecule 4-1BB, which has recently been reported to modulate HBV-specific CD8 T-cell functions in the liver.[28,29]

At Week 3, after protein prime vaccination, there was no significant difference in the expression levels of PD-1, LAG-3, and TOX (Fig. 6A) on C_{93}-specific CD8$^+$ T cells or in the frequencies of cells expressing PD-1 or co-expressing PD-1 and LAG-3 or TOX between low- and high-titer HBV carriers (Fig. 6B). Starting at Week 5 after the MVA boost, however, the expression of PD-1, LAG-3, and TOX on C_{93}-specific CD8$^+$ T cells increased considerably in high-titer carriers, correlating temporally with their loss of cytokine production. Moreover, the fraction of C_{93}-specific CD8$^+$ T cells positive for one or more of these molecules remained significantly elevated in high-titer carriers, whereas their frequencies gradually decreased in the livers of low-titer mice and were barely detectable on liver-infiltrating MVA-specific CD8$^+$ T cells (Fig. S3). Interestingly, we found that the co-stimulatory molecule 4-1BB was also significantly upregulated on C_{93}-specific CD8$^+$ T cells in high-titer carriers following the complete vaccination scheme, with kinetics resembling those observed for the co-inhibitory markers (Fig. 6C). Moreover, most 4-1BB$^+$ $C_{93}{}^+$ CD8$^+$ T cells co-expressed PD-1 (Fig. 6D).

Taken together, our results indicate that vaccine-induced CD8$^+$ T cells exposed to high levels of HBV antigens in the liver overexpress markers associated with T-cell dysfunction, thereby suppressing their antiviral function and preventing them from eliminating HBV-infected hepatocytes.

Discussion

High HBV antigen levels remain the major obstacle to successful therapeutic vaccination, as they suppress vaccine-elicited CD8 T-cell responses, thereby limiting the antiviral effect. For the first time, we demonstrate that immunization with our clinical candidate therapeutic vaccine, *TherVacB*, could induce HBV-specific CD8$^+$ T cells in the presence of high HBV loads as efficiently as in low-level persistent infection. Although vaccine-elicited CD8$^+$ T cells in high-titer HBV carriers initially displayed some antiviral efficacy, they remained trapped in the liver, progressively became dysfunctional, and were ultimately eliminated. Loss of CD8 T-cell effector functions in high-titer HBV carriers correlated with overexpression of checkpoint inhibitors, such as PD-1, exhaustion-related transcription factor TOX, and co-stimulatory molecule 4-1BB.

CD44$^+$Cxcr6$^+$ CD8$^+$ T cells. (H) Frequencies of splenic C_{93}-specific effector and effector memory (CD44$^+$CD62L$^-$) and central memory (CD44$^+$CD62L$^+$) CD8$^+$ T cells in low-titer mice. Statistical analyses were performed using two-way ANOVA. Asterisks mark statistically significant differences: *$p \leq 0.05$; **$p \leq 0.01$; ***$p \leq 0.001$; ****$p \leq 0.0001$. Data are presented as mean and SEM (n ≥4). MFI, mean fluorescent index; n.a., not applicable; TherVacB, therapeutic hepatitis B vaccine.

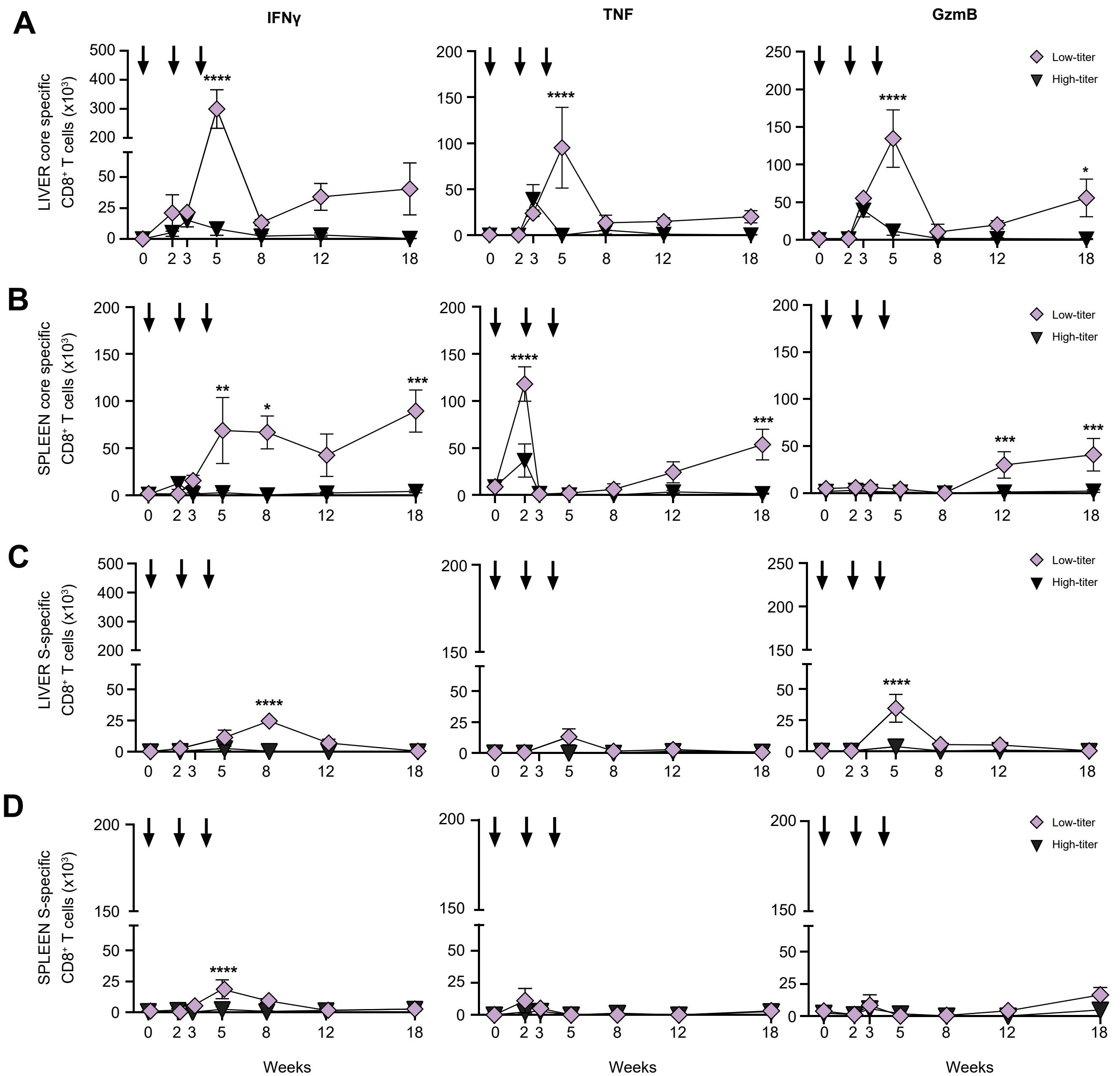

Fig. 5. Effect of *TherVacB* on the functionality of HBV-specific CD8$^+$ T cells. High- and low-titer HBV carriers were immunized with *TherVacB*, as shown in Fig. 1A; analyses were performed at the indicated time points. Numbers of (A) liver-associated and (B) splenic core-specific IFNγ^+, TNF$^+$, and GzmB$^+$ CD8$^+$ T cells; (C) liver-associated and (D) splenic S-specific IFNγ^+, TNF$^+$, and GzmB$^+$ CD8$^+$ T cells detected by intracellular cytokine staining after 16-h stimulation with corresponding HBV S- or core-derived peptide pools. Statistical analyses were performed using two-way ANOVA. Asterisks mark statistically significant differences: *$p \leq 0.05$; **$p \leq 0.01$; ***$p \leq 0.001$; ****$p \leq 0.0001$. Data are presented as mean and SEM own (n ≥4). GzmB, granzyme B; IFNγ, interferon γ; *TherVacB*, therapeutic hepatitis B vaccine; TNF, tumor necrosis factor.

This comprehensive and longitudinal study clearly defines a period during which vaccine-induced HBV-specific CD8 T cells lose effector function, potentially informing the timing of additional immunostimulatory therapies.

HBV-specific CD8$^+$ T cells constitute a functionally and phenotypically heterogeneous population that varies with antigen specificity.[30] We found that the *TherVacB*-elicited core-specific CD8 T-cell response displayed dynamic changes over time in quantity, function, and signature, which significantly differed between low- and high-titer HBV carrier mice and closely correlated with the antiviral effect of the vaccination. By contrast, the S-specific CD8 T-cell response remained much less prominent than the core-specific CD8 T-cell response and, surprisingly, did not depend on the initial HBV titer. This implies that core-specific CD8$^+$ T cells play a key role in clearing persistent HBV infection following *TherVacB* in our

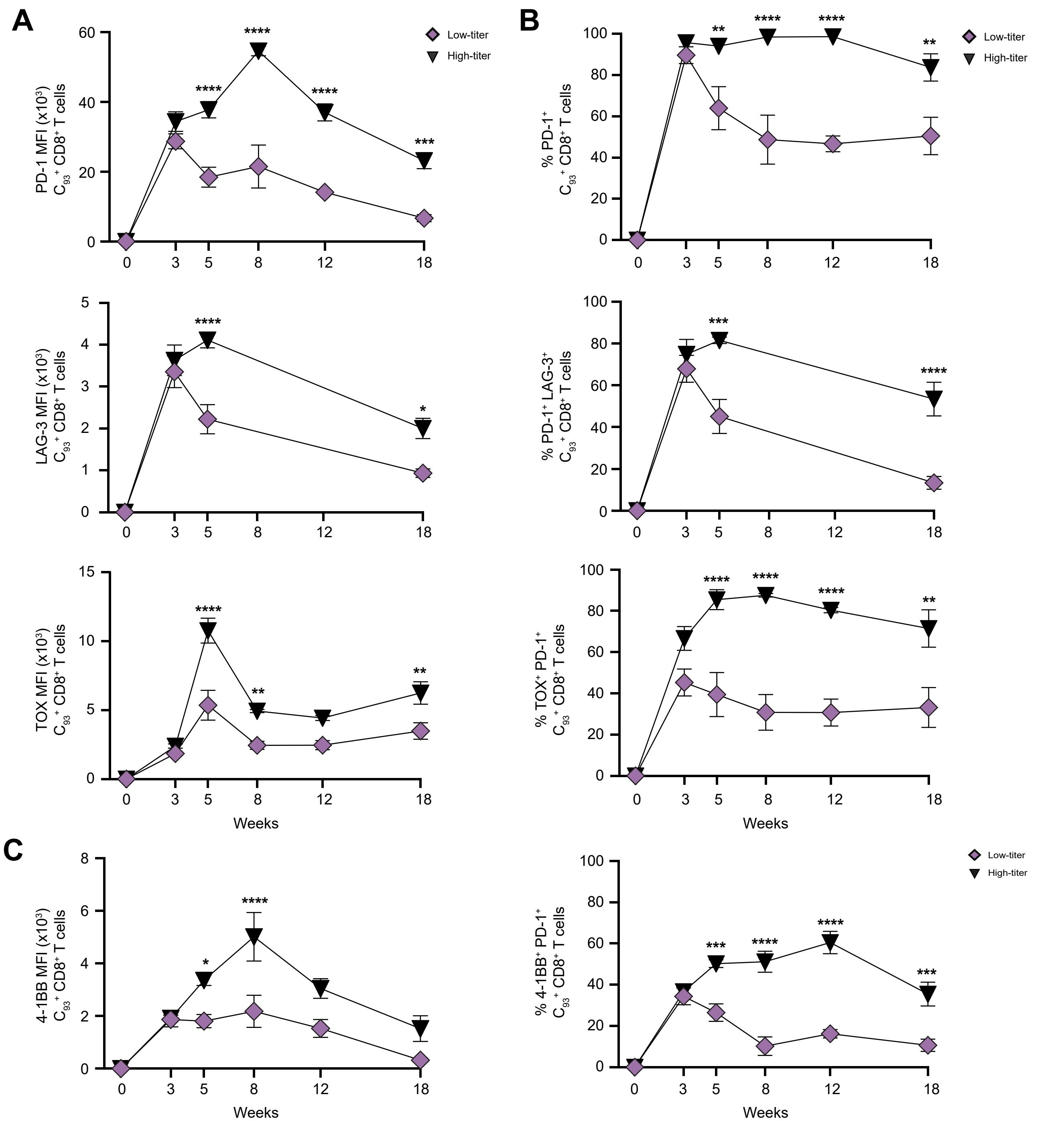

Fig. 6. Expression of immune checkpoint markers on *TherVacB*-induced CD8$^+$ T cells. High- and low-titer HBV carriers were immunized with *TherVacB*, as shown in Fig. 1A; analyses were performed at the indicated time points. (A) MFIs of PD1, Lag3, and TOX and (B) frequencies of C_{93}-specific liver-associated PD-1$^+$, PD-1$^+$TOX$^+$, and PD-1$^+$LAG-3$^+$ CD8$^+$ T cells. (C) MFI of 4-1BB and frequencies of C_{93}-specific liver-associated 4-1BB$^+$ PD1$^+$ CD8$^+$ T cells. Statistical analyses were performed using two-way ANOVA. Asterisks mark statistically significant differences: *p ≤0.05; **p ≤0.01; ***p ≤0.001; ****p ≤0.0001. Data are presented as mean and SEM (n ≥4). MFI, mean fluorescent index; *TherVacB*, therapeutic hepatitis B vaccine.

mouse model. It has been previously demonstrated that the HBV S protein is less immunogenic and less accessible to CD8$^+$ T cells than the core protein.[14] The disparity between S- and core-specific CD8 T-cell responses observed in this study may also be a result of increased presentation of immunodominant core-derived epitope in the livers of C57BL/6J mice replicating HBV of genotype D. Chronic exposure to the antigen can lead to the functional impairment or deletion of antigen-specific CD8$^+$ T cells.[31] Our data demonstrate that the presence of high HBV antigen does not prevent the induction

of virus-specific $CD8^+$ T cells or inhibit their antiviral functions during protein immunizations with *TherVacB*. The booster vaccination with MVA, specifically designed to stimulate and expand HBV-specific CD8 T-cell responses,[9,10] was a pivotal moment determining the outcomes of the therapeutic vaccination. In low-titer carriers, the MVA boost led to rapid expansion of liver-associated core-specific effector $CD8^+$ T cells, which correlated with the elimination of persistent HBV infection. Interestingly, these highly functional $Cxcr6^+$ $CD8^+$ T cells detected in the livers of low-titer mice during and after HBV clearance resembled, in their characteristics, a distinct population of protective liver-resident memory (T_{RM}) $CD8^+$ T cells, which were also described in patients with immune control of chronic hepatitis B.[32] Although the MVA boost resulted in strong upregulation of Cxcr6 on vaccine-induced $CD8^+$ T cells in high-titer carriers, these cells failed to control HBV infection and completely lost their effector functions. In addition, without efficient elimination of HBV-positive hepatocytes, a rebound in circulating HBsAg levels was observed in high-titer mice despite the presence of anti-HBs antibodies, which were of comparable quantity and neutralization capacity to those detected in low-titer mice. Simultaneous detection of HBsAg and neutralizing anti-HBs antibodies was previously reported in patients immunized with a therapeutic vaccine containing Pre-S1, Pre-S2, and S antigens but lacking a HBV-specific $CD8^+$ T-cell response.[16] This underlines the role of vaccine-induced cytotoxic $CD8^+$ T cells in reducing HBsAg reservoir in the liver and sustaining functional HBV cure.

Our previous studies have clearly shown that the antiviral effect of therapeutic vaccination depends strictly on the amount of antigen presented in the liver.[7,12,13] We assume that increased antigen recognition in the livers of high-titer mice provided additional stimulatory signals, which in turn 'shut down' the antiviral immune response. Moreover, a strong elevation of Cxcr6 on core-specific $CD8^+$ T cells in the livers of high-titer carriers, as compared with low-titer carriers, could act as a 'trap' for activated $CD8^+$ T cells, preventing their homing to the spleens. Instead, vaccine-elicited cells progressively perished in the liver of high-titer carriers, resembling the scenario of patients with chronic hepatitis B who demonstrate only very scarce S-specific $CD8^+$ T cells after long-term exposure to high levels of HBsAg.[33]

There is increasing evidence that high HBV antigen load is a key determinant limiting the efficacy of therapeutic vaccination, not only in mouse models[12,34,35] but also in patients with chronic hepatitis B.[36] Chronic HBV carriers, who display HBsAg levels over 1,000 IU/ml, would most likely require a combination of therapeutic vaccination with antiviral drugs or immunomodulators to achieve HBV control. We previously reported that a combination of specific siRNAs and *TherVacB* could induce a sustained antiviral effect in high-titer mice, owing to enhanced functionality of vaccine-induced $CD8^+$ T cells and low expression of checkpoint markers.[12] More recently, high antigen burden was shown to drive rapid $CD8^+$ T cell dysfunction, with increased PD-1 expression and reduced cytokine production in AAV-HBV mice.[37] Here, we demonstrate that almost all *TherVacB*-elicited $CD8^+$ T cells in high-titer carriers not only continuously upregulated PD-1 but also co-expressed high levels of TOX, a transcription factor associated with persistent antigen stimulation and the severity of T-cell dysfunction in chronic hepatitis B.[27] TOX causes epigenetic changes to maintain the exhausted state in virus-specific $CD8^+$ T cells.[38] This epigenetic imprinting implies that once vaccine-elicited $CD8^+$ T cells express TOX, they may not regain function, even with further treatments. This supports our previous findings that combinatorial treatment of *TherVacB* with siRNA targeting PD-L1 allowed for a more sustained HBV control when applied during priming rather than boosting of *TherVacB*[13] and may also explain the low efficacy of PD-1 blockade in patients during boost vaccination.[39] Thus, we propose that additional immune modulation during therapeutic vaccination should be applied early, before CD8 T-cell function is lost.

Recent evidence suggests that dysfunction of HBV-specific $CD8^+$ T cells during chronic infection may result from several novel mechanisms that differ from classical exhaustion. Virus-specific $CD8^+$ T cells can be attenuated through (i) increased TGF-β signaling;[40] (ii) upregulation of co-stimulatory TNF receptors,[29] particularly 4-1BB; and (iii) a liver-immune rheostat, which enhances activity of transcription factor CREM and inhibits T-cell receptor signaling.[28] We found that significantly elevated expression of 4-1BB on liver-associated vaccine-elicited $CD8^+$ T cells in high-titer carriers closely correlated in time with a loss of cytokine production. Expression of 4-1BB may not only directly influence the function of vaccine-elicited HBV-specific $CD8^+$ T cells in the liver but may also suggest involvement of the immune rheostat, as 4-1BB is one of the main effector molecules regulated by CREM.[28] This suggests that targeting 4-1BB may be a promising approach to enhance the efficacy of therapeutic vaccination in high-titer HBV infection. Moreover, most of the $4\text{-}1BB^+$ $CD8^+$ T cells also co-expressed PD-1, suggesting that multiple mechanisms contribute to the dysfunction of HBV-specific $CD8^+$ T cells in high-titer carriers.

In conclusion, our study elucidates how high HBV antigen load suppresses antiviral CD8 T-cell immunity after therapeutic vaccination, providing valuable insights for the design of more effective immunotherapeutic strategies against chronic hepatitis B.

Affiliations

[1]*Institute of Virology, School of Medicine and Health, Technical University of Munich/Helmholtz Munich, Munich, Germany;* [2]*German Center for Infection Research (DZIF), Partner Site Munich, Munich, Germany;* [3]*Institute of Pathology, School of Medicine and Health, Technical University of Munich, Munich, Germany;* [4]*Institute for Medical Microbiology, Immunology and Hygiene, Technical University of Munich, Munich, Germany;* [5]*Institute of Molecular Immunology and Experimental Oncology, School of Medicine and Health, Technical University of Munich, TUM University Hospital Rechts der Isar, Munich, Germany*

Abbreviations

AAV, adeno-associated virus; ALT, alanine aminotransferase; anti-HBc, hepatitis B core antibody; anti-HBe, hepatitis B e antibody; anti-HBs, hepatitis B surface antibody; ARRIVE, Animal Research: Reporting of *in vivo* Experiments; c-di-AMP, bis-(3′,5′)-cyclic dimeric adenosine monophosphate; CTLA4, cytotoxic T-lymphocyte associated protein 4; Cxcr6, C-X-C motif chemokine receptor 6; FELASA, Federation of Laboratory Animal Science Associations; GV-SOLAS, German regulations of the Society for Laboratory Animal Science; GzmB,

granzyme B; ICS, intracellular cytokine staining; IFNγ, interferon γ; LAG-3, lymphocyte-activation gene 3; LAL, liver-associated lymphocyte; MVA, modified vaccinia Ankara; NK, natural killer; PD-1, programmed cell death 1; pgRNA, pregenomic RNA; sALT, serum alanine transaminase; *TherVacB*, therapeutic hepatitis B vaccine; TNF, tumor necrosis factor; TOX, thymocyte selection-associated high-mobility group box; Treg, regulatory T cell; VLP, virus-like particle.

Financial support

This study was funded by the German Research Foundation (DFG) via SFB-TRR179 (project no. 272983813 to UP and AK), the PoC initiative by the Helmholtz and Fraunhofer Associations (to UP), the EU Horizon 2020 consortium TherVacB (to UP), the German Center for Infection Research (DZIF) via project TTU 05.803 'HBV Cure' (to UP), the German Ministry for Education and Research (BMBF) via the TherVacB PLUS project, C-NATM—Cluster for Nucleic Acid Therapeutics Munich funded in the Clusters4Future initiative (funding no. 03ZU1201BA to UP), and SFB-TRR338 (project no. 452881907 to DB and UP). EAO and MG received a PhD scholarship from the Turkish Ministry of Education. HAK received an MD scholarship from DZIF. JS was supported by the DZIF Academy's maternity leave program. The funders had no role in the study design, data collection, analysis, or interpretation, the writing of the manuscript, or the decision to publish the results.

Conflicts of interest

UP and AK are named as inventors on a patent application (PCT/EP2017/050553) describing the therapeutic vaccination scheme for TherVacB and the vector MVA-HBVac. UP is a scientific founder, shareholder, and board member of SCG Cell Therapy and serves as an *ad hoc* advisor to Aligos, Arbutus, GSK, Roche, Sanofi, Vaccitech/Barinthos, and Vir Bio. The remaining authors declare no conflicts of interest.

Please refer to the accompanying ICMJE disclosure forms for further details.

Authors' contributions

Designed the study: EAO, UP, AK. Performed the experiments: EAO, AK, JS, MG, MT, NM, HAK, SS. Analyzed the data: EAO, AK. Provided essential reagents and assistance: CM, KS, DB, PK. Provided supervision and funding: AK, UP. Wrote and finalized the manuscript: EAO, UP, AK. Read and approved the final version of the manuscript: all authors.

Data availability

Data supporting the findings of this study are available within the article and its supplementary materials. The raw data may be available from the corresponding author upon reasonable request.

Acknowledgements

The authors acknowledge Susanne Miko, Theresa Asen, and Philipp Hagen for their excellent technical support. We thank the Comparative Experimental Pathology (CEP) of the TUM School of Medicine and Health for their excellent technical support. We thank Noomen Hamad and Anna Hochholzer for generating HBV-specific multimers and TarGet Laboratory and the ViVeM Center/UMR 1089 Platform (Nantes Université, INSERM, France) for the production of the AAV-HBV.

Supplementary data

Supplementary data to this article can be found online at https://doi.org/10.1016/j.jhepr.2026.102033.

References

Author names in bold designate shared co-first authorship

[1] World Health Organization. Hepatitis B. n.d. https://www.who.int/news-room/fact-sheets/detail/hepatitis-b. [Accessed 2 June 2025].
[2] Kosinska AD, Bauer T, Protzer U. Therapeutic vaccination for chronic hepatitis B. Curr Opin Virol 2017;23:75–81.
[3] Fanning GC, Zoulim F, Hou J, et al. Therapeutic strategies for hepatitis B virus infection: towards a cure. Nat Rev Drug Discov 2019;18:827–844.
[4] Protzer U, Maini MK, Knolle PA. Living in the liver: hepatic infections. Nat Rev Immunol 2012;12:201–213.
[5] Gehring AJ, Protzer U. Targeting innate and adaptive immune responses to cure chronic HBV infection. Gastroenterology 2019;156:325–337.
[6] Bertoletti A, Gehring AJ. The immune response during hepatitis B virus infection. J Gen Virol 2006;87:1439–1449.
[7] Kosinska AD, Festag J, Mück-Häusl M, et al. Immunogenicity and antiviral response of therapeutic hepatitis B vaccination in a mouse model of HBeAg-negative, persistent HBV infection. Vaccines 2021;9:841.
[8] **Backes S**, **Jäger C**, Dembek CJ, et al. Protein-prime/modified vaccinia virus Ankara vector-boost vaccination overcomes tolerance in high-antigenemic HBV-transgenic mice. Vaccine 2016;34:923–932.
[9] Su J, Brunner L, Ates Oz E, et al. Activation of CD4 T cells during prime immunization determines the success of a therapeutic hepatitis B vaccine in HBV-carrier mouse models. J Hepatol 2023;78:717–730.
[10] Kosinska AD, Kächele M, Kerth HA, et al. MVA-HBVac—a novel vaccine vector that allows pan-genotypic targeting of hepatitis B virus by therapeutic vaccination. Mol Ther Nucleic Acids 2025;36:102641.
[11] Kosinska AD, Moeed A, Kallin N, et al. Synergy of therapeutic heterologous prime-boost hepatitis B vaccination with CpG-application to improve immune control of persistent HBV infection. Sci Rep 2019;9:1–10.
[12] **Michler T**, **Kosinska AD**, Festag J, et al. Knockdown of virus antigen expression increases therapeutic vaccine efficacy in high-titer hepatitis B virus carrier mice. Gastroenterology 2020;158:1762. 75.e9.
[13] Bunse T, Kosinska AD, Michler T, Protzer U. PD-L1 Silencing in liver using siRNAs enhances efficacy of therapeutic vaccination for chronic hepatitis B. Biomolecules 2022;12:470.
[14] Fumagalli V, Lucia P Di, Venzin V, et al. Serum HBsAg clearance has minimal impact on $CD8^+$ T cell responses in mouse models of HBV infection. J Exp Med 2020;217:e20200298.
[15] Michler T, Zillinger J, Hagen P, et al. The lack of HBsAg secretion does neither facilitate induction of antiviral T cell responses nor hepatitis B virus clearance in mice. Antivir Res 2024;226:105896.
[16] Ji Y, Le Bert N, Lai-Hung Wong G, et al. The impact of hepatitis B surface antigen reduction via small interfering RNA treatment on natural and vaccine (BRII-179)-induced hepatitis B virus-specific humoral and cellular immune responses. Gastroenterology 2025;169:136–149.
[17] **Sacherl J**, **Kosinska AD**, Kemter K, et al. Efficient stabilization of therapeutic hepatitis B vaccine components by amino-acid formulation maintains its potential to break immune tolerance. JHEP Rep 2023;5:100603.
[18] Effenberger M, Stengl A, Schober K, et al. FLEXamers: a double tag for universal generation of versatile peptide-MHC multimers. J Immunol 2019;202:2164–2171.
[19] Desmet VJ, Gerber M, Hoofnagle JH, et al. Classification of chronic hepatitis: diagnosis, grading and staging. Hepatology 1994;19:1513–1520.
[20] Kerth HA, Kächele M, Müller N, et al. Therapeutic hepatitis B vaccine employing DNA prime–MVA boost scheme requires additional priming with recombinant HBsAg to elicit an adequate antibody response. Front Immunol 2026;17:1771887.
[21] Wettengel JM, Linden B, Esser K, et al. Rapid and robust continuous purification of high-titer hepatitis B virus for in vitro and in vivo applications. Viruses 2021;13:1503.
[22] Rust LN, Wettengel JM, Biswas S, et al. Liver-specific transgenic expression of human NTCP in rhesus macaques confers HBV susceptibility on primary hepatocytes. Proc Natl Acad Sci U S A 2025;122:e2413771122.
[23] Chisari FV, Isogawa M, Wieland SF. Pathogenesis of hepatitis B virus infection. Pathol Biol (Paris) 2010;58:258–266.
[24] Tse SW, Radtke AJ, Espinosa DA, et al. The chemokine receptor CXCR6 is required for the maintenance of liver memory $CD8^+$ T cells specific for infectious pathogens. J Infect Dis 2014;210:1508–1516.
[25] Pallett LJ, Maini MK. Liver-resident memory T cells: life in lockdown. Semin Immunopathol 2022;44:813–825.
[26] Ma J, Zheng B, Goswami S, et al. $PD1^{Hi}$ $CD8^+$ T cells correlate with exhausted signature and poor clinical outcome in hepatocellular carcinoma. J Immunother Cancer 2019;7:33.
[27] Heim K, Neumann-Haefelin C, Thimme R, et al. Heterogeneity of HBV-specific CD8+ T-cell failure: implications for immunotherapy. Front Immunol 2019;10:2240.
[28] **Bosch M**, **Kallin N**, Donakonda S, et al. A liver immune rheostat regulates CD8 T cell immunity in chronic HBV infection. Nature 2024;631:867–875.
[29] **Andreata F**, **Laura C**, **Ravà M**, et al. Therapeutic potential of co-signaling receptor modulation in hepatitis B. Cell 2024;187:4078. 94.e21.

[30] Hoogeveen RC, Robidoux MP, Schwarz T, et al. Phenotype and function of HBV-specific T cells is determined by the targeted epitope in addition to the stage of infection. Gut 2018:893–904.
[31] Fisicaro P, Barili V, Montanini B, et al. Targeting mitochondrial dysfunction can restore antiviral activity of exhausted HBV-specific CD8 T cells in chronic hepatitis B. Nat Med 2017;23:327–336.
[32] Pallett LJ, Davies J, Colbeck EJ, et al. IL-2^{high} tissue-resident T cells in the human liver: sentinels for hepatotropic infection. J Exp Med 2017;214:1567–1580.
[33] Le Bert N, Gill US, Hong M, et al. Effects of Hepatitis B surface antigen on virus-specific and global T cells in patients with chronic hepatitis B virus infection. Gastroenterology 2020;159:652–664.
[34] Lewis G, Malo K, Rowland T, et al. IL-2 and IL-15 augment HBV therapeutic vaccination and PD1 blockade for functional cure in the AAV-HBV mouse model. Front Immunol 2025;16:1562107.
[35] **Makrinos D**, **Sun Y**, Davis H, et al. A multivalent mRNA-LNP therapeutic vaccine with broad cross-genotype immunogenicity elicits clearance of HBV infected hepatocytes. BioRxiv 2025. https://doi.org/10.64898/2025.12.03.692117.
[36] Mak LY, Lok ASF. Update on combination therapies against HBV in clinical investigations. Antivir Res 2026;245:106321.
[37] Rexhouse C, Bailey JT, Moshkani S, et al. High antigen burden drives $CD8^+$ T cell dysfunction in a mouse model of chronic hepatitis B virus infection. J Virol 2025;99:e0071125.
[38] Alfei F, Kanev K, Hofmann M, et al. TOX reinforces the phenotype and longevity of exhausted T cells in chronic viral infection. Nature 2019;571:265–269.
[39] Gane E, Verdon DJ, Brooks AE, et al. Anti-PD-1 blockade with nivolumab with and without therapeutic vaccination for virally suppressed chronic hepatitis B: a pilot study. J Hepatol 2019;71:900–907.
[40] **Heim K**, **Sagar**, Sogukpinar Ö, et al. Attenuated effector T cells are linked to control of chronic HBV infection. Nat Immunol 2024;25:1650–1662.

Keywords: HBV-specific $CD8^+$ T cells; Chronic hepatitis B; CD8 T-cell dysfunction; Therapeutic vaccination; *TherVacB*; Antigen-induced T-cell dysfunction.
Received 25 February 2026; received in revised form 1 September 2026; accepted 5 September 2026; Available online 10 September 2026

Journal of Hepatology, Volume *8*

Supplemental information

High HBV antigen levels drive loss of vaccine-induced CD8$^+$ T-cell function in carrier mice over time

Edanur Ates Öz, Jinpeng Su, Merve Gültan, Margaret Tulessin, Nikolas Müller, Hélène Anne Kerth, Swati Singh, Carolin Mogler, Katja Steiger, Jochen Wettengel, Dirk Busch, Percy Knolle, Ulrike Protzer, and Anna Kosinska

High HBV antigen levels drive loss of vaccine-induced CD8$^+$ T-cell function in carrier mice over time

Edanur Ates Öz, Jinpeng Su, Merve Gültan, Margaret Tulessin, Nikolas Müller, Hélène Anne Kerth, Swati Singh, Carolin Mogler, Katja Steiger, Jochen M. Wettengel, Dirk H. Busch, Percy A. Knolle, Ulrike Protzer, Anna D. Kosinska

Table of contents

Fig. S1……………………………………………………………………………….2

Fig. S2……………………………………………………………………………….3

Fig. S3……………………………………………………………………………….4

Table S1……………………………………………………………………………..5

Table S2……………………………………………………………………………..6

Table S3……………………………………………………………………………..6

Table S4……………………………………………………………………………..7

Table S5……………………………………………………………………………..7

Supplementary Figures

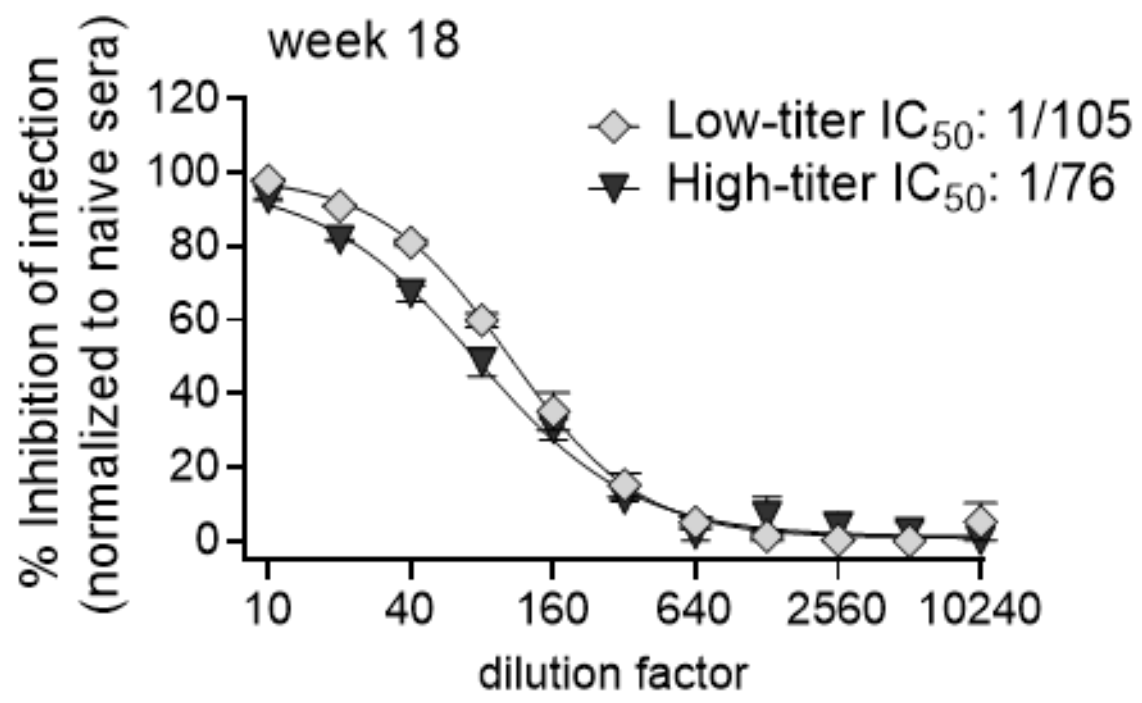

Fig. S1: Serum neutralization assay at week 18

C57BL/6J mice were intravenously injected with AAV-HBV1.2 six weeks before vaccination to establish low- and high-level persistent HBV replication. Mice were vaccinated intramuscularly following the *TherVacB* regimen. The analyses were performed at week 18, 14 weeks after completion of the vaccination scheme. The percent inhibition of infection by sera from low- and high-titer mice was determined by a neutralization assay using recombinant HBV expressing NanoLuc luciferase. IC_{50} values determined from neutralization curves are shown. The mean and SEM are shown (n = 5).

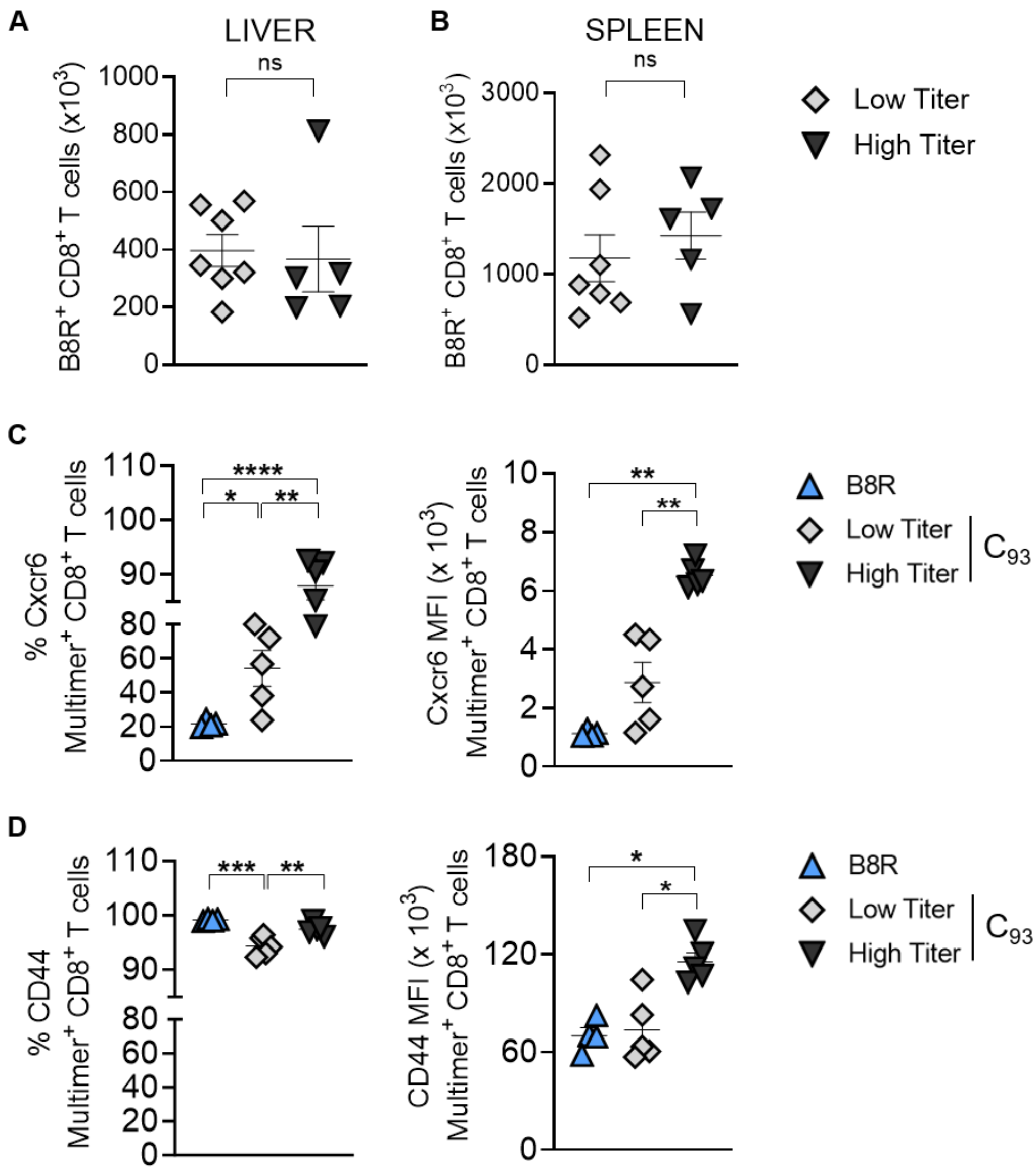

Fig. S2. MVA-specific $CD8^+$ T cells in the liver and spleen at week 5

C57BL/6J mice were intravenously injected with AAV-HBV1.2 six weeks before vaccination to establish low- and high-level persistent HBV replication. Mice were vaccinated intramuscularly following the *TherVacB* regimen. The analyses were performed 1 week after completion of the vaccination scheme, at week 5. Counts of MVA(B8R)-specific (A) liver-associated and (B) splenic $CD8^+$ T cells in low- and high-titer mice. Expression of (C) Cxcr6 and (D) CD44 on B8R- and C_{93}-specific liver-associated $CD8^+$ T cells and their mean fluorescent index (MFI) at week 5. Cell counts were determined using CountBright™ absolute counting beads for flow cytometry after ex vivo multimer staining. Statistical analyses were performed using (A-B) the Mann-Whitney U-test and (C-D) the two-way ANOVA. Asterisks mark statistically significant differences: *$p \leq 0.05$; **p≤0.01, ***p≤0.001; ****p≤0.0001; n.s, not significant. The mean and SEM are shown (n ≥ 4).

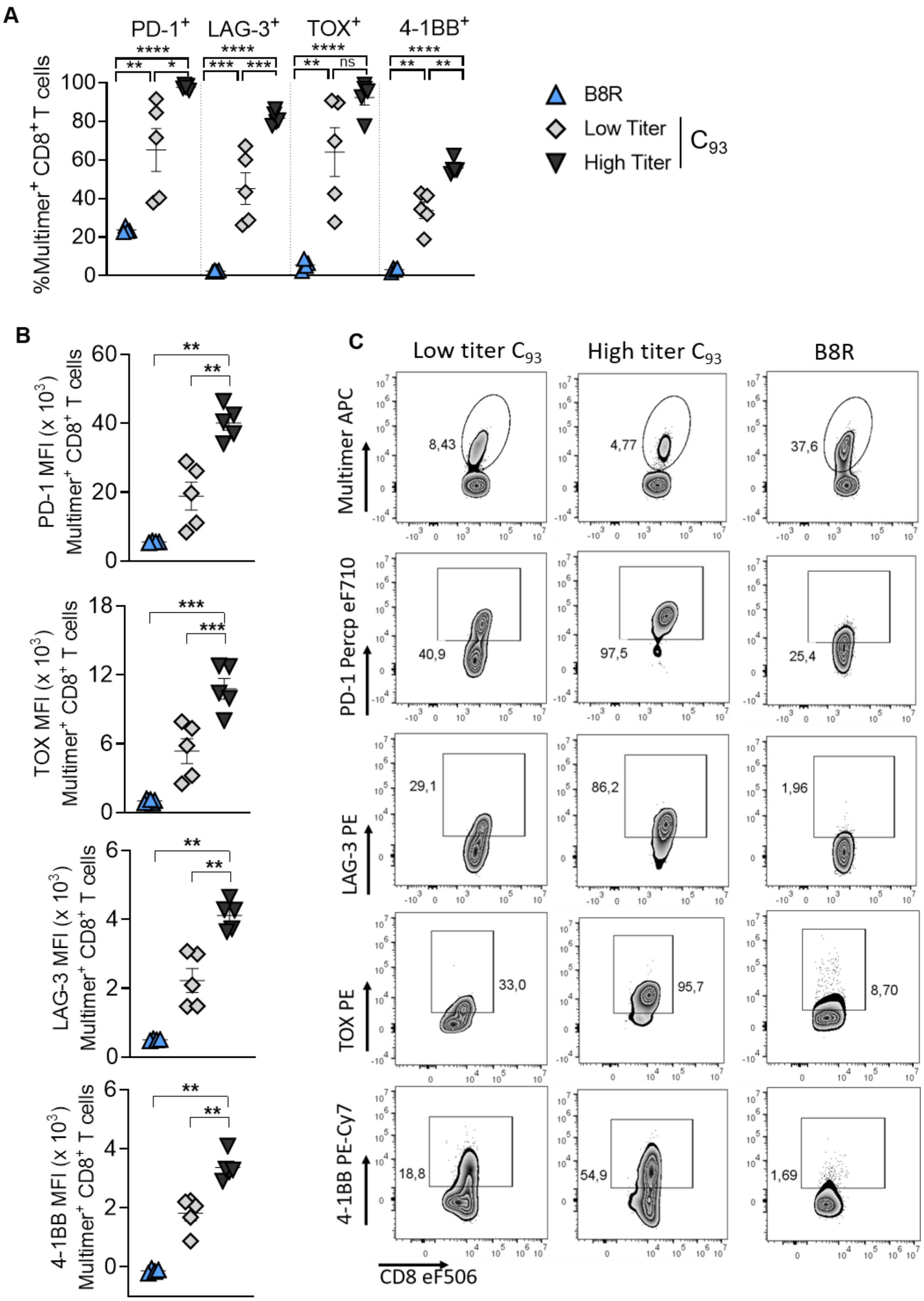

Fig. S3. Comparison of MVA-specific and HBV-specific immune cell marker expression on CD8+ T cells at week 5

C57BL/6J mice were intravenously injected with AAV-HBV1.2 six weeks before vaccination to establish low- and high-level persistent HBV replication. Mice were vaccinated intramuscularly

following the *TherVacB* regimen. The analyses were performed 1 week after completion of the vaccination scheme, at week 5. (A-B) Expression of PD-1, LAG-3, TOX, and 4-1BB on MVA(B8R)-specific and C_{93}-specific liver-associated $CD8^+$ T cells and their mean fluorescent index (MFI) at week 5. Cell counts were determined using CountBright™ absolute counting beads for flow cytometry after ex vivo multimer staining. (C) Representative flow cytometry plots of C_{93}-specific and B8R-specific and $CD8^+$ T cells detected with C_{93}- or B8R-specific MHC-I in isolated liver-associated lymphocytes. Statistical analyses were performed using the two-way ANOVA. Asterisks mark statistically significant differences: **p*≤0.05; **p≤0.01, ***p≤0.001; ****p≤0.0001; n.s: not significant. The mean and SEM are shown (n ≥ 4).

Supplementary Materials and Methods

Table S1: Antibodies

Antibody	Conjugated Color	Dilution	Reference Number	Major Supplier
anti-hGzmB	PE	1:100	GRB04	Invitrogen
anti-m4-1BB	PE-Cy7	1:200	25-1371-82	eBioScience
anti-mCD19	AF700	1:100	115528	Biolegend
anti-mCD25	APC	1:100	17-0251-81	eBioscince
anti-mCD3	FITC	1:100	100203	Biolegend
anti-mCD4	AF700	1:100	100536	Biolegend
anti-mCD4	PE	1:200	12-0041-83	eBioscience
anti-mCD4	APC	1:100	17-0041-84	eBioscience
anti-mCD44	PE	1:250	12-0441-82	eBioscience
anti-mCD45.2	PE	1:200	12-0454-83	eBioscience
anti-mCD8a	APC	1:100	17-0041-83	Invitrogen
anti-mCD8a	eF506	1:100	69-0081-82	Invitrogen
anti-mCD8a	Pacific Blue	1:100	558106	BD Biosciences
anti-mCxcr6	FITC	1:100	151108	Biolegend
anti-mFoxP3	FITC	1:100	11-5773-82	Invitrogen
anti-mIFNγ	FITC	1:300	554411	BD Biosciences
anti-mLag3	PE	1:200	12-2231-83	Invitrogen
anti-mLag3	eFluor450	1:100	48-2231-80	eBioscience

anti-mNK1.1	PE-Cy7	1:100	108714	Biolegend
anti-mPD1	PerCp eFluor 710	1:200	46-9985-82	eBioscience
anti-mTNF	PE-Cy7	1:200	557644	BD Biosciences
anti-mTox	PE	1:200	12-6502-82	Invitrogen
Fixable Viability Dye	eFluor780	1:3000	65-0865-18	eBioscience
Strep-Tavidin	APC	1:100	17-4317-82	Invitrogen

Table S2: HBsAg-derived peptide pool

Numbering	Position	Amino acid Sequence
Sp3-37	145-159	GNCTCIPIPSSWAFA
Sp3-38	149-163	CIPIPSSWAFAKYLW
Sp3-39	153-167	PSSWAFAKYLWEWAS
Sp3-40	157-171	AFAKYLWEWASARFS
Sp3-41	161-175	YLWEWASARFSWLSL
Sp3-42	165-179	WASARFSWLSLLVPF
Sp3-43	169-183	RFSWLSLLVPFVQWF
Sp3-44	173-187	LSLLVPFVQWFVGLS
Sp3-45	177-191	VPFVQWFVGLSPTVW
Sp3-46	181-195	QWFVGLSPTVWLSAI
Sp3-47	185-199	GLSPTVWLSAIWMMW
Sp3-48	189-203	TVWLSAIWMMWYWGP
Sp3-49	193-207	SAIWMMWYWGPSLYS
Sp3-50	197-211	MMWYWGPSLYSIVSP
Sp3-51	201-215	WGPSLYSIVSPFIPL
Sp3-52	205-219	LYSIVSPFIPLLPIF
Sp3-53	209-223	VSPFIPLLPIFFCLW
Sp3-54	213-226	IPLLPIFFCLWVYI

Table S3: HBcAg-derived peptide pool

Numbering	Position	Amino acid Sequence
Cp2-188	70-87	TWVGGNLEDPISRDLVVS
Cp2-189	77-94	EDPISRDLVVSYVNTNMG
Cp2-190	84-101	LVVSYVNTNMGLKFRQLL
Cp2-191	91-108	TNMGLKFRQLLWFHISCL
Cp2-192	98-115	RQLLWFHISCLTFGRETV
Cp2-193	105-122	ISCLTFGRETVIEYLVSF
Cp2-194	112-129	RETVIEYLVSFGVWIRTP
Cp2-195	119-136	LVSFGVWIRTPPAYRPPN

Cp2-196	126-143	IRTPPAYRPPNAPILSTL
Cp2-197	133-150	RPPNAPILSTLPETTVVR
Cp2-198	140-157	LSTLPETTVVRRRGRSPR

Table S4: Set of primers and cycling conditions for the HBV-DNA PCR

Primer Name	Sequence
rcDNA Fwd (HBV1745)	5’-GGAGGGATACATAGAGGTTCCTTGA-3’
rcDNA Rev (HBV 1844)	5´-GTTGCCCGTTTGTCCTCTAATTC-3´
mPrp Fwd	5’- GCGGTACATGTTTTCACGGTAGTA-3’
mPrp Rev	5’-GAGCAGGCCCATGATCCA-3’

Cycling conditions: 5 min 95 °C followed by 40× (15 s 95 °C, 10 s 60 °C, 25 s 72°C).

Table S5: The set of primers and cycling conditions for HBV-RNA PCR

Primer Name	Sequence
pgRNA Fwd[1]	5’-GAGTGTGGATTCGCACTCC-3’
pgRNA Rev[1]	5’-GAGGCGAGGGAGTTCTTCT-3’
mGAPDH Fwd[2]	5’-ACCAACTGCTTAGCCC-3’
mGAPDH Rev[2]	5’-CCACGACGGACACATT-3’

Cycling conditions[1]: 5 min 95 °C followed by 40× (15 s 95 °C, 30 s 60 °C).

Cycling conditions[2]: 5 min 95 °C followed by 40× (15 s 95 °C, 10 s 60 °C, 25 s 72 °C).
