# Supplementary material for: High HBV antigen levels drive loss of vaccine-induced CD8+ T-cell function in carrier mice over time
Source: JHEP Rep. 2026 Sep 10;8(12):102033. doi: 10.1016/j.jhepr.2026.102033 (PMC13743956; doi:10.1016/j.jhepr.2026.102033)
Supplement: Supplementary file 4 [file mmc2.docx]

**JHEP Reports**

**CTAT methods**

Tables for a “Complete, Transparent, Accurate and Timely account” (CTAT) are now mandatory for all revised submissions. The aim is to enhance the reproducibility of methods.

- Only include the parts relevant to your study
- Refer to the CTAT in the main text as ‘Supplementary CTAT Table’
- Do not add subheadings
- Add as many rows as needed to include all information
- Only include one item per row

**If the CTAT form is not relevant to your study, please outline the reasons why:**

1.1 **Antibodies**

| **Name** | **Citation** | **Supplier** | **Cat no.** | **Clone no.** |
| --- | --- | --- | --- | --- |
| anti-hGzmB PE | - | Invitrogen | GRB04 | GB11 |
| anti-m4-1BB Pe-Cy7 | - | eBioScience | 25-1371-82 | 17B5 |
| anti-mCD19 AF700 | - | Biolegend | 115528 | 6D5 |
| anti-mCD25 APC | - | eBioscince | 17-0251-81 | PC61.5 |
| anti-mCD3 FITC | - | Biolegend | 100203 | 17A2 |
| anti-mCD4 AF700 | - | Biolegend | 100536 | RM4-5 |
| anti-mCD4 PE | - | eBioscience | 12-0041-83 | GK1.5 |
| anti-mCD4 APC | - | eBioscience | 17-0041-84 | GK1.5 |
| anti-mCD44 PE | - | eBioscience | 12-0441-82 | IM7 |
| anti-mCD45.2 PE | - | eBioscience | 12-0454-83 | 104 |
| anti-mCD8a APC | - | Invitrogen | 17-0081-82 | 53-6.7 |
| anti-mCD8a eF506 | - | Invitrogen | 69-0081-82 | 53-6.7 |
| anti-mCD8a PB | - | BD Biosciences | 558106 | 53-6.7 |
| anti-mCxcr6 FITC | - | Biolegend | 151108 | SA051D1 |
| anti-mFoxP3 FITC | - | Invitrogen | 11-5773-82 | FJK-16s |
| anti-mIFNγ FITC | - | BD Biosciences | 554411 | XMG1.2 |
| anti-mLag3 PE | - | Invitrogen | 12-2231-83 | C9B7W |
| anti-mLag3 eF450 | - | eBioscience | 48-2231-80 | C9B7W |
| anti-mNK1.1 PE-Cy7 | - | Biolegend | 108714 | PK136 |
| anti-mPD1 PerCp-eF710 | - | eBioscience | 46-9985-82 | J43 |
| anti-mTNF PE-Cy7 | - | BD Biosciences | 557644 | MP6-XT22 |
| anti-mTox PE | - | Invitrogen | 12-6502-82 | TXRX10 |

1.2 **Cell lines**

| **Name** | **Citation** | **Supplier** | **Cat no.** | **Passage no.** | **Authentication test method** |
| --- | --- | --- | --- | --- | --- |
| HepG2-NTCP | - | In-house production | - | P15-20 | IF staining using Myrcludex B staining |

1.3 **Organisms**

| **Name** | **Citation** | **Supplier** | **Strain** | **Sex** | **Age** | **Overall n number** |
| --- | --- | --- | --- | --- | --- | --- |
| Mouse (Mus musculus) | - | Janvier Labs (Le Genest-Saint-Isle, France) | C57BL/6JRj | Male | 8-10 weeks old | 140 |

1.4 **Sequence based reagents**

| **Name** | **Sequence** | **Supplier** |
| --- | --- | --- |
| rcDNA Fwd (HBV1745) | 5’-GGAGGGATACATAGAGGTTCCTTGA-3’ | MicroSynth AG, Switzerland |
| rcDNA Rev (HBV 1844) | 5´-GTTGCCCGTTTGTCCTCTAATTC-3´ | MicroSynth AG, Switzerland |
| mPrp Fwd | 5’- GCGGTACATGTTTTCACGGTAGTA-3’ | MicroSynth AG, Switzerland |
| mPrp Rev | 5’-GAGCAGGCCCATGATCCA-3’ | MicroSynth AG, Switzerland |
| pgRNA Fwd | 5’-GAGTGTGGATTCGCACTCC-3’ | MicroSynth AG, Switzerland |
| pgRNA Rev | 5’-GAGGCGAGGGAGTTCTTCT-3’ | MicroSynth AG, Switzerland |
| mGAPDH Fwd | 5’-ACCAACTGCTTAGCCC-3’ | MicroSynth AG, Switzerland |
| mGAPDH Rev | 5’-CCACGACGGACACATT-3’ | MicroSynth AG, Switzerland |

1.5 **Biological samples**

| **Description** | **Source** | **Identifier** |
| --- | --- | --- |
| Liver-associated lymphocytes and splenocytes isolated from AAV-HBV-infected mouse | In-house (mice originally sourced from Janvier Labs) | permission number: ROB-55.2-2532.Vet_02-18-24 |
| Serum collected from AAV-HBV-infected mice | In-house (mice originally sourced from Janvier Labs) | permission number: ROB-55.2-2532.Vet_02-18-24 |

1.6 **Deposited data**

| **Name of repository** | **Identifier** | **Link** |
| --- | --- | --- |
| **-** | **-** | **-** |

1.7 **Software**

| **Software name** | **Manufacturer** | **Version** |
| --- | --- | --- |
| FlowJo software | Tree Star, USA | 10.8 |
| GraphPad Prism | GraphPad Software Inc., USA | 9.5 or 10.1 |

1.8 **Other (*e.g*. drugs, proteins, vectors etc.)**

| AAV-HBV | vector | TarGet Laboratory and the ViVeM Center/UMR 1089 Platform (Nantes Université, INSERM, France) |
| --- | --- | --- |
| c-di-AMP | adjuvant | InvivoGen, USA |
| HBsAg-VLPs (genotype A) | protein | Biovac, South Africa |
| HBcAg-VLPs(genotype D) | protein | Lativijas Biomedicinas, Latvia |
| MVA-S | vector | In-house produced |
| MVA-core | vector | In-house produced |

1.9 **Please provide the details of the corresponding methods author for the manuscript:**

Dr. Edanur Ates Öz, Institute of Virology, Technical University of Munich, Trogerstr. 30 81675 Munich, Germany, Phone: +49 89 4140 6826, email: [edanur.ates-oz@tum.de](mailto:edanur.ates-oz@tum.de)

Dr. Anna D. Kosinska, Institute of Virology, Technical University of Munich, Trogerstr. 30 81675 Munich, Germany, Phone: +49 89 4140 7359, email: [anna.kosinska@tum.de](mailto:anna.kosinska@tum.de)

**2.0	Please confirm for randomised controlled trials all versions of the clinical protocol are included in the submission. These will be published online as supplementary information.**

**N/A**
